# Supplementary material for: Graph2GO: a multi-modal attributed network embedding method for inferring protein functions
Source: Gigascience. 2020 Aug 8;9(8):giaa081. doi: 10.1093/gigascience/giaa081 (PMC7414417; doi:10.1093/gigascience/giaa081)
Supplement: giaa081_GIGA-D-19-00430_Revision_1 [file giaa081_giga-d-19-00430_revision_1.pdf]

GigaScience

Graph2GO: a multi-modal attributed network embedding method for inferring protein functions

--Manuscript Draft--

Manuscript Number:	GIGA-D-19-00430R1
Full Title:	Graph2GO: a multi-modal attributed network embedding method for inferring protein functions
Article Type:	Technical Note
Funding Information:	National Cancer Institute (P30CA016058) — Not applicable
Abstract:	Background: Identifying protein functions is important for many biological applications. Since experimental functional characterization of proteins is time-consuming and costly, accurate and efficient computational methods for predicting protein functions are in great demand for generating testable hypotheses guiding large-scale experiments. Results: Here we proposed Graph2GO, a multi-modal graph-based representation learning model that can integrate heterogeneous information including multiple types of interaction networks (sequence similarity network and protein-protein interaction network) and protein features (amino acid sequence, subcellular location and protein domains) to predict protein functions on Gene Ontology. Comparing Graph2GO to BLAST, a baseline model and two popular protein function prediction methods: Mashup and deepNF, we demonstrated that our model can achieve state-of-the-art performance. We show the robustness of our model by testing on multiple species. We also provide a web server supporting function query and downstream analysis on-the-y. Conclusions: Graph2GO is the rst model that utilized attributed network representation learning methods to model both interaction networks and protein features for predicting protein functions, and achieved promising performance. Our model can be easily extended to include more protein features to further improve the performance. Besides, Graph2GO is also applicable to other application scenarios involving biological networks and the learned latent representations can be used as feature inputs for machine learning tasks in various downstream analysis.
Corresponding Author:	Yan Zhang<br>Ohio State University<br>Columbus, Ohio UNITED STATES
Corresponding Author Secondary Information:	
Corresponding Author's Institution:	Ohio State University
Corresponding Author's Secondary Institution:	
First Author:	Kunjie Fan
First Author Secondary Information:	
Order of Authors:	Kunjie Fan<br>Yuanfang Guan<br>Yan Zhang
Order of Authors Secondary Information:	
Response to Reviewers:	Response letter (We also provide a PDF version in the submission)<br><br>We thank the editors and reviewers for their insightful comments and suggestions and believe that after addressing each comment the manuscript is stronger. We have highlighted all our answers in red color.<br><br>> Editor's comments:

Powered by Editorial Manager® and ProduXion Manager® from Aries Systems Corporation

> Both reviewers point out several issues with the evaluation and comparison procedures, which may have introduced bias. Before making a decision on a revised manuscript we will seek further advice from our reviewers and I urge you to make an effort to fully address their comments.

Authors' answer: We appreciate the feedback. We have worked carefully to solve the problem concerning evaluation and comparison procedures that might introduce bias. We have added a new evaluation metric F-max that is widely used by the community to replace our previous F1-score that is only based on top three predictions to make the evaluation more reliable. We include the detailed definition of these evaluation metrics so that general readers can understand. We also elaborate more on our DNN classification model and the models we compare with as suggested by reviewers. For experiments, we have added more details to clarify any confusions. Besides, we make changes to get rid of any variations that might impede the comparability of the results. We also followed other suggestions carefully and explained how we addressed them in the following point-to-point response.

>Reviewer #1's comments:
>
>This work describes a new method that uses attributed network representation that uses both interaction networks and protein features to predict protein function, when the function are represented as GO terms. This method of integrating heterogeneous information is, to my knowledge, novel and interesting. The methodology and results are mostly well described (but see comments below).

>Major comments:
>My main concern is the rather low F1 scores when compared with the apparent better performance of the AUPR scores, as shown in Table 1. (Incidentally, of showing F1 scores, why not show average precision and recall so that the reader can establish the contribution of the precision and recall to the F1 score?) The F1 scores are quite low (0.29 for CC and MF, and 0.07 for BP). I believe the authors could explain that, especially in light of the overall good performance in Table 2. Again, Figure 3 shows a low performance of F1 (0.25-0.35) of F1. How does that work with the F1 of 0.717 reported in Table 2?

Authors' answer: Thanks for pointing out this problem. We should apologize for the inconsistence of the use of F1-score. In Table 1, Table 3 and Figure 3, the F1-score we used is only based on top three predictions, as used by Mashup and deepNF that we want to compare with. In Table 2, when comparing with BLAST, we used the common F1-score, since F1-score based on top three predictions cannot be calculated for BLAST. The F1-score based on top three predictions tend to be small across all models, compared to other metrics. In order to solve the inconsistence, we decide to change the evaluation metric to F-max, the maximum F1-score over all possible thresholds. This evaluation metric is also adopted by CAFA (Critical Assessment of protein Function Annotation algorithms). The detailed definition of this metric along with others are introduced in section "Evaluation metric" under "Results". Looking at the results based on F-max, the performance is more consistent with other metrics, and our model is still better than other methods under this metric.

>I could not find definitions for m-AUPR and M-AUPR. It is my understanding that micro-averaged area under the precision-recall curve (m-AUPR) is computed by first vectorizing and then computing the AUPR; Macro-AUPR (M-AUPR) is computed by first computing the AUPR for each function separately, and then averaging these values across all functions. I would like to know how this was implemented here, and how the differences in performance are explained. While these performance metrics may be well-understood in the machine learning community, I think that the authors should elaborate a bit more on the choice of these metrics and their meaning, as Gigascience aims at a broader audience than those well-versed in machine learning.

Authors' answer: Thanks for pointing out this issue. We have added a new section "Evaluation metric" under "Results" to elaborate on the metrics we used. Since the protein function prediction is a multi-label task, we need to evaluate the performance on all labels (GO terms). One of the most popular metrics used in this setting is macro-

or micro- area under receiver operating characteristic (ROC) curves. However, given the fact that the dataset is kind of skewed (more negative samples than positive samples), the precision-recall curve will give a more informative picture of an algorithm's performance than ROC curves, as pointed by Davis and Goadrich (reference 42 in the manuscript). Therefore, we adopt micro-AUPR (m-AUPR) and macro-AUPR (M-AUPR) as two evaluation metrics. m-AUPR is computed by first vectorizing all labels (consider as one label) and then computing the AUPR, while M-AUPR is computed by first calculating AUPR for each label individually and then performing the unweighted mean of all AUPRs. Both of the metrics can properly represent the overall performance of a multi-label classifier.

>In Table 2, the authors show that the overall performance of Graph2GO is good, when compared with a baseline of BLAST. It does seem however, that the BLAST performance is rather high. CAFA typically reports Fmax (not F1) values between 0.4 and 0.5. I am wondering how such high BLAST F1 score was achieved.
One possible issue may be the random split of training and testing sets as reported in P 6 "Comparison with BLAST". A random split does not ensure that there are no homologs for the training set in the test set, which may mean that the authors are testing on part of their training set and test set. The authors should ensure that any sequence in the test set is not too similar to the sequences in the training set.E.g.
https://urldefense.proofpoint.com/v2/url?u=https-3A__www.biorxiv.org_content_10.1101_626507v4.full&d=DwIGaQ&c=k9MF1d71ITtkuJx-PdWme51dKbmfPEvxwt8SFEkBfs4&r=ekJXXuaVVww1UTqb5FGVAqKm1D8j8S7Zredd9hAkY7k&m=0Feecqdx0RHOBGTTz1ex_w6u3ZYkDPiddrOHjdLNzoE&s=NMlQXdy4qrXzxCszFk_3ASxxc-3QmA6j1BxmSisYeLM&e=

Authors' answer: Thanks for pointing out this question. We have added a new comparison situation where all training samples sharing more than 50% identity with one of the test samples are removed. Therefore, we ended up with two datasets: (1) full dataset that uses all randomly split training samples; (2) partial dataset that removes potential homologs. We can see that the results on the partial dataset are now similar to the results from CAFA. From the updated Table 2, we can see that Graph2GO is more robust than BLAST given the fact that Graph2GO shows smaller drop than BLAST from evaluation under full dataset condition to partial dataset condition. As a result, Graph2GO outperforms BLAST even more under the partial dataset condition across all ontologies. The detailed results and analysis are in section "Comparison with BLAST" and Table 2.

>Minor comments:
>
>Figure 3 is too small to be legible.

Authors' answer: Thanks for pointing out the problem. We have updated Figure 3 for the updated evaluation metrics and made the figure clearer this time.

>Table 3: seemingly low F1 performances, per organism.

Authors' answer: The results of each organism is consistent with the results on human, even for the low F1 score. As we answered to the first point, the low F1 score is due to the definition of our previously used F1 (based only on top three predictions). We have changed this type of F1 score to F-max, as defined in the section "Evaluation metrics". The updated results still seem consistent with the results on human.

>Reviewer #2's comments:
>
>This paper presents an approach to predicting protein function that leverages heterogenous sources of knowledge about proteins, including relational information derived from protein interactions and sequence similarity relationships. The authors have clearly presented their computational architecture, which is interesting and as far as I know represents a novel contribution.
However, I have some concerns. My main concerns with this manuscript are (a) a number of (apparent) variations in experimental settings that impeded comparability of

results and might suggest some cherry-picking, and (b) the lack of comparison with the tasks and data sets used in the community evaluation CAFA (Critical Assessment of Function Annotation). Together, it means it is difficult to fully judge the contributions of the work.
>
>Major point 1:
>
>- Few details of the DNN are provided; this should be elaborated.

Authors’ answer: Thanks for pointing out this problem. We have added more details about DNN in section “Deep neural network classifier” to make it clearer.

>- Is the CNN used in the 'Feature transfer' experiments the same as the DNN of the original experiments? If not, what are the differences, and why is the same algorithm not used for comparability? Is retraining needed in any case, can't the comparison of the sparsity vs non-sparsity group be done as an analysis over the results data? I don't feel that this is explained well.

Authors’ answer: Thanks for raising this question. The CNN model used in “Feature transfer” experiment is not the same the DNN we used in the second part of our Graph2GO model. We have added a detailed description of this model in the supplementary material named “Convolutional Neural Networks (CNNs)”.

The hypothesis we want to claim here is to show Graph2GO, a model making use of feature transfer within the network, is better at dealing with proteins without sufficient features due to the annotation bias. In other words, we want to show that the performance on the sparsity group is not a lot worse than that of on the non-sparsity group, when using Graph2GO. That’s why we use the ratio between the performance on sparsity group and non-sparsity group as a measure. We want to compare with a method without using the concept of feature transfer. Actually, we have many choices, for example, SVM, logistic regression, DNN or CNN. The reason why we chose CNN is that CNN performs better than DNN or SVM in this case since it is good at extracting features. But we can also choose other methods to compare with, no matter the method itself is good or not, since what we care about is the ratio of performance between the sparsity group and non-sparsity group.

The re-training is not needed for Graph2GO in this analysis, since we just analyzed the results data by dividing the test results into two groups and calculated the corresponding metrics.

>- In the comparison with deepNF and Mashup, the authors do not explain how the embeddings produced by these methods are used. The authors have explained that these methods produce embeddings that are analogous to the embeddings derived by the authors, but then do not use the same approach in the subsequent stages of the classification process; they use a multi-layer NN for Graph2Go and an SVM for the other two methods. For direct comparability, the overall architecture for learning would be kept the same, and only the input embeddings would be changed. How else can we know whether the key improvement comes from the embedding approach, or the classification model? The current comparison does not appear to be like-for-like.

Authors’ answer: Thanks for pointing out this issue. We previously touched a little bit on these two methods in the fourth paragraph in the “Introduction”. We have added a few more descriptions of these two methods.

We previously used SVM for these methods as the classification model because they used SVM in their original implementation. In order to make it more comparable, we have changed their classification model to DNN as well, with the same architecture of Graph2GO. We have updated Figure 3 accordingly with new comparison results, and we have shown that our method outperforms other two methods In CC and MF ontologies, and achieve comparable results in BP ontology.

>- In the comparison across species, the authors do not explain their experimental framework sufficiently. Do they control for overlap (very high sequence similarity) with the human training data they have used in developing their model? Do they 'hide'/hold

Powered by Editorial Manager® and ProduXion Manager® from Aries Systems Corporation

back annotations [all, or just some?] for the proteins in these data sets? Furthermore, they claim "decent" performance (compared to what?) and yet the performance on these data sets is far lower than on the other test data they present. This does not at all 'prove' the robustness; it needs to be explained.

Authors’ answer: Thanks for pointing out this problem. In the comparison across species, we are actually performing experiments in each species individually. That’s to say, for each species, we download their data, and then performing cross-validation within this species, just like what we did for human dataset. We didn’t work in a transfer learning manner, training on human data and test on other species.

By conducting experiments in multiple species, we want to see whether the performance is consistent among all species to show the robustness of our method. We have also included the results on human dataset in the Table 3, and we can see that the performance of these six species are consistently decent, except that the results on s. cerevisiae and bacillus subtilis in BP ontology are a lot better than those of other species.

>Major point 2:
>
>There have been a number of studies over several years that explore methods for automatic assignment of protein function (CAFA4 has just started). The authors cite some of this work (Reference 1) but then go on to claim that homology-based methods are still the norm. That was true 10 years ago, but I am not convinced that it is still true today. Importantly, data sets have been prepared for CAFA, as well as many methods, and the authors clearly need to discuss how their work relates to that work. Indeed, I would suggest that without a direct comparison to the data sets used in CAFA, it is not possible for the authors to claim state-of-the-art performance. BLAST is still a meaningful baseline method to compare with, but it is certainly not representative of the state-of-the-art.
This comparison may not be entirely feasible given that CAFA test sets are large and only a fraction of them are used for the final evaluation, but IMO it is important to explore this and at least attempt to come close to a similar experimental framework to test the authors' approach. At a minimum, the authors should be using the same metrics for evaluation as CAFA -- perhaps in addition to their own metrics -- to enable meaningful comparison, and of course discussing results on that task in relation to theirs. A comparison that only considers embedding methods is incomplete, in particular given that there are several methods used in CAFA that explicitly support integration of multiple sources of information, including relational information (see [1] for instance).

Authors’ answer: Thanks for the great suggestions. For the evaluation metrics, we use similar metrics with CAFA: F-max, macro-AUPR and micro-AUPR. We have added the section “Evaluation metrics” under “Results” to describe the metrics in detail. AUROC is used for term-centric evaluation in CAFA. Here we adopt AUPR instead, since as suggested by Davis and Goadrich (reference 42 in the manuscript), when the dataset is highly skewed, which is the case in this protein function prediction task, AUPR is a more informative metric than AUROC.

Mashup and deepNF are both state-of-the-art network-based embedding methods for predicting protein functions by integrating multiple network information. By comparing with these two methods, we have shown that Graph2GO is among the state-of-the-art network-based embedding methods for protein function prediction. We agree that it is ideal to compare with a larger pool of methods, including integrative methods not based on embedding in CAFA. However, we feel that given the scope of the study, we would like to focus on several methods that are recently published and well received in the community. We would like to continue the development and comparison in the lines of CAFA in the future, which may need our own implementations of CAFA methods that require devotion of more time. We think participating in a large-scale competition like CAFA in the future might be beneficial to our method comparison and development.

In summary, our method not only outperforms Mashup and deepNF in performance, but also exhibits some improvements in the method design. For example, Graph2GO

Powered by Editorial Manager® and ProduXion Manager® from Aries Systems Corporation

can consider both heterogenous network structural information and protein attributes (including sequences, locations and protein domains) while other two methods only consider network features. Besides, the concept of feature transfer adopted by Graph2GO is advantageous for solving the annotation bias, which is a novel contribution.

>On metrics, the use of the groupings of GO terms based on sparsity of annotations is not well justified; I do not understand what this represents, in particular given that the number of proteins annotated to a given GO term is not reflective of any biological reality -- it simply reflects areas of study and our current level of biological understanding. GO is organised hierarchically along semantic/ontological lines, and the authors do not appear to recognise this characteristic. Furthermore, there exists a grouping of GO terms called GO Slims which already seeks to reduce the number of classes that should be considered [2] (now called subsets:https://urldefense.proofpoint.com/v2/url?u=http-3A__geneontology.org_docs_go-2Dsubset-2Dguide_&d=DwIGaQ&c=k9MF1d71ITtkuJx-PdWme51dKbmfPEvxwt8SFEkBfs4&r=ekJXXuaVVww1UTqb5FGVAqKm1D8j8S7Zredd9hAkY7k&m=0Feecqdx0RHOBGTTz1ex_w6u3ZYkDPiddrOHjdLNzoE&s=J9pWUSCW_9XLhgcFjbPLOw2acB-nA9wZrNl5fqLnx44&e=).

Authors’ answer: Thanks for raising this question. We group GO terms based on the sparsity of annotations to assess the impact of annotation bias on function prediction, and to show the performance of our method in more detail. Typically, the more data we have (meaning the annotations are less sparse), the better the function prediction model can perform. By dividing the GO terms in groups with different number of annotated proteins and analyzing the results separately, we can show how our model works when the data is sparse or when the data is abundant.

As for GO Slims, we found that they are grouped mainly based on different species. Since we already analyze the results for each species individually, we feel it is better to stay with the original GO groupings. Thanks for the suggestions.

>In addition, why are only the top three predictions considered for evaluation purposes, in calculating F1-score? Having an absolute number, rather than based on confidence/probability etc. requires justification.

Authors’ answer: Thanks for pointing out this problem. We have changed this metric to F-max as suggested. The definition is provided in section “Evaluation metrics”. Previously we used F1-score based on top three predictions since it was used by Mashup and deepNF, and we adopted F1-score as well for the comparison. Now we use F-max to compare all these methods, which is reasonable and more widely used.

>In the methods, the authors do not adequately explain their output representation -- is it framed as a multi-label classification problem, or as a multi-class problem (i.e. can a single input have multiple labels in the output)? Is it just a flat set of GO terms, so that the semantic interdependencies between the terms are not considered? (I later found this detail in the Results; it should come earlier.) It is actually a hierarchical classification problem [3].
This hierarchical nature also interacts with metrics and evaluation; please see [4] and [5]. The authors should consider/discuss their evaluation protocols in this context.

Authors’ answer: Thanks for raising this issue. Our task is a multi-label problem, we mentioned that in section “Deep neural network classifier” under “Methods”. Our output representation is a flat set of GO terms. In our current implementation, we didn’t consider the semantic interdependencies between the terms, which might cause inconsistency between the prediction of leaf GO terms and the corresponding parent GO terms. However, when we generate the dataset for model training and testing, we do assign the parent GO term to the protein when the child GO term is present. We think this will partially solve the issue of incomplete GO term annotations in the dataset. Besides, since we report a probability in range [0, 1] for each GO term, the users can decide the reliability of the GO term (no matter leaf or parent GO term) by the probability.

Powered by Editorial Manager® and ProduXion Manager® from Aries Systems Corporation

	We have also added some discussion about how we can treat this as a hierarchical classification problem in the future under the “Discussion” section. In the future, one way to treat it as a hierarchical classification is to add the hierarchical constraints in the output layer to force the predictions of leaf nodes are consistent with the predictions of the corresponding parent nodes. Another potential method is to explicitly add the GO hierarchy into the model, along with our other network components to inform the training process, since we can regard the GO hierarchy as a network. We will leave this integration to the future work.<br><br>>In the section of "Graph2Go pipeline" the authors mention two alternative architectures. Ideally, they would test both architectures (possibly moving the detail to Supplementary) and indicate which worked better.<br><br>Authors’ answer: Thanks for pointing out this problem. We have added the comparison in the supplementary material. From the comparison, we see that the model that trains independent VGAE for each network and combines their embeddings is better than the model that first combines the networks and train one VGAE to obtain the overall embeddings. This result supports our Graph2GO architecture.<br><br>>On language; the writing is in general very good. There are a few colloquialisms that could be replaced: "plenty of" -> "substantial"; "really good" -> "very good" or "excellent". "noises" should just be "noise". "well suitable" should be "well suited". I found at least one typo "nueral".<br><br>Authors’ answer: Thanks for pointing these out. We have corrected the language problems.
**Additional Information:**	
**Question**	**Response**
Are you submitting this manuscript to a special series or article collection?	No
**Experimental design and statistics**<br><br>Full details of the experimental design and statistical methods used should be given in the Methods section, as detailed in our Minimum Standards Reporting Checklist. Information essential to interpreting the data presented should be made available in the figure legends.<br><br>Have you included all the information requested in your manuscript?	Yes
**Resources**<br><br>A description of all resources used, including antibodies, cell lines, animals and software tools, with enough information to allow them to be uniquely identified, should be included in the Methods section. Authors are strongly encouraged to cite Research Resource	Yes

Identifiers (RRIDs) for antibodies, model organisms and tools, where possible.<br><br>Have you included the information requested as detailed in our Minimum Standards Reporting Checklist?	
**Availability of data and materials**<br><br>All datasets and code on which the conclusions of the paper rely must be either included in your submission or deposited in publicly available repositories (where available and ethically appropriate), referencing such data using a unique identifier in the references and in the “Availability of Data and Materials” section of your manuscript.<br><br>Have you have met the above requirement as detailed in our Minimum Standards Reporting Checklist?	Yes

Click here to access/download;Manuscript;Gigascience_Graph2GO_v2_hig

GigaScience, 2017, 1–11

doi: xx.xxxx/xxxx
Manuscript in Preparation
Paper

PAPER

Graph2GO: a multi-modal attributed network embedding method for inferring protein functions

Kunjie Fan[1], Yuanfang Guan[2,*] and Yan Zhang[1,3,*]

[1]Department of Biomedical Informatics, College of Medicine, The Ohio State University, Columbus, OH 43210, United States of America and [2]Department of Computational Medicine and Bioinformatics, University of Michigan, Ann Arbor, MI 48109, United States of America and [3]The Ohio State University Comprehensive Cancer Center (OSUCCC - James), Columbus, OH 43210, United States of America

[*]**Correspondence address.** Yuanfang Guan, Department of Computational Medicine and Bioinformatics, University of Michigan, Ann Arbor, MI 48109, United States of America. E-mail: gyuanfang@umich.edu; Yan Zhang, Department of Biomedical Informatics, College of Medicine, The Ohio State University, Columbus, OH 43210, United States of America. E-mail: Yan.Zhang@osumc.edu

Abstract

Background: Identifying protein functions is important for many biological applications. Since experimental functional characterization of proteins is time-consuming and costly, accurate and efficient computational methods for predicting protein functions are in great demand for generating testable hypotheses guiding large-scale experiments. **Results**: Here we proposed Graph2GO, a multi-modal graph-based representation learning model that can integrate heterogeneous information including multiple types of interaction networks (sequence similarity network and protein-protein interaction network) and protein features (amino acid sequence, subcellular location and protein domains) to predict protein functions on Gene Ontology. Comparing Graph2GO to BLAST, a baseline model and two popular protein function prediction methods: Mashup and deepNF, we demonstrated that our model can achieve state-of-the-art performance. We show the robustness of our model by testing on multiple species. We also provide a web server supporting function query and downstream analysis on-the-fly. **Conclusions**: Graph2GO is the first model that utilized attributed network representation learning methods to model both interaction networks and protein features for predicting protein functions, and achieved promising performance. Our model can be easily extended to include more protein features to further improve the performance. Besides, Graph2GO is also applicable to other application scenarios involving biological networks and the learned latent representations can be used as feature inputs for machine learning tasks in various downstream analysis.

Key words: protein function prediction; graph neural network; attributed network embedding; representation learning; multi-modal model

Background

Knowledge of protein functions is of great importance to understand life at a molecular level, study disease mechanisms and help explore novel therapeutic targets. However, experimental identification of protein functions are time-consuming and expensive, which are not suitable for large-scale applications. Therefore, high-throughput computational methods are required for discovering protein functions with reasonable quality and accuracy [1], and provide testable hypotheses for targeted experimental validation.

Most existing computational algorithms exploit homology inference to infer protein functions [2, 3, 4], which are based on the assumption that proteins with similar sequences frequently carry out similar functions. A standard approach is simply to transfer annotations from the best-annotated BLAST hit [5]. Some approaches involve the use of protein-protein interaction (PPI) networks based on the fact that proteins closer

Compiled on: April 15, 2020.
Draft manuscript prepared by the author.

in the network have greater chance to share similar functions [6, 7]. Some other methods utilize domain content in the sequence to assign functions [8, 9]. Under the assumption that domains are protein structural architecture modules, it makes sense that protein function should be closely related to or determined by the composite domains. There are also other methods making use of other sources of information for protein function prediction, such as protein subcellular localization [10, 11], post-translational modifications [11] and literature [12].

Given the limited prediction capacity of a single source of information, many methods have been proposed to combine several kinds of information and take advantage of the power of machine learning techniques. For instance, INGA [13] performs sequence similarity and domain architecture searches, and combines them with enrichment analysis on interaction networks to derive the consensus prediction. Similarly, COFACTOR [14] consists of three individual pipelines for sequence-, structure- and PPI-based predictions by querying UniProt-GOA [15], BioLip and STRING [16] databases respectively and generates the consensus based on three confidence scores obtained from the three pipelines. DeepGO [17] uses representation learning methods to learn features from sequence and interaction networks respectively and then combine them to predict the function using a deep neuro-symbolic model. Although these methods could achieve reasonable prediction accuracy, one limitation is that they only treat multiple kinds of features separately and do not consider the relationships between proteins for each feature, which might result in the loss of informaiton contained within the interactions. One possible solution to take into account the relationships is to use protein-protein interaction networks to transfer features between proteins.

Graphs, such as those representing social networks, molecular graph structures [18] and biological protein-protein interaction networks, occur naturally in various real-world scenarios and have important applications in modern machine learning. Modeling the interactions between entities as graphs has enabled researchers to understand the network system in a systematic manner. For example, in a social network application one might wish to predict the role of a person or recommend new friends to a user [19]; in a clinical application, researchers want to predict new therapeutic applications of drug molecules [20]; and basic scientists are also interested in classifying the roles of a protein in a biological interaction graph [21]. Mashup [22] is a framework for scalable and robust multiple network integration and generates low-dimensional vectors for nodes by characterizing topological context in heterogeneous PPI networks based on the random walk with restart method. The key of Mashup is the use of random walk with restart method to analyze the network structure. They also proposed novel methods for dimension reduction and integration of heterogeneous networks by solving optimization problems. DeepNF [23] is a network fusion method for extracting protein features from multiple heterogeneous interaciton networks based on multimodal deep auto-encoders. DeepNF also utilizes the random walk with restart to extract information about network structure for individual PPI networks. DeepNF proposed a different integration method based on auto-encoders. Both of these two methods adopt a two-stage model: first generating informative embeddings based on network structures in an unsupervised manner, then building a supervised classification model to predict GO terms with embeddings as the input. Although both Mashup and DeepNF can generate embeddings used for downstream protein function prediction, they only consider topological information contained in multiple PPI networks while ignoring informative protein attributes such as protein sequence or protein domains, thus might lack important information.

A crucial challenge in machine learning on graphs is finding a way to incorporate multiple types of information about the structure and attributes of the graph into the machine learning model. Traditional approaches usually use summary graph statistics [24], kernel functions [25] or handcrafted features to represent local neighborhood structures. Nowadays, people seek to learn representations that encode structural information in a data-driven way. The idea behind these representation learning methods is to learn a function that maps nodes in the graph to points in a low-dimensional vector space. By optimizing this mapping the geometric relationships in the learned space could reflect the original structure of the graph, and the learned representation can be used as feature inputs for downstream machine learning applications [26]. These network representation learning methods can also make use of node attributes to generate more informative embeddings, such as user profiles in a social network and protein signatures in a protein interaction network [21].

In this paper, we propose Graph2GO, a multi-modal graph-based architecture that can make use of several kinds of data sources in a unified way to predict protein function. Unlike other consensus methods we mentioned earlier, we first use protein-protein interactions and sequence similarities to build two graphs separately and use protein sequence information, protein subcellular location, protein domains or any other possible information as node attributes in both graphs. Given the attributed graph, we use the attributed network representation learning algorithm to obtain informative embeddings for each node in each graph, i.e., each protein. In this way, we manage to learn representations by modeling both node attributes and network structures comprehensively and simultaneously. Then we combine these two embeddings and use them to predict the protein functions with a feedforward neural network model.

As far as we know, we are the first to use attributed network representation learning methods to model both interaction network and sequence similarity network with node attributes and predict protein functions, and we have successfully achieved state-of-the-art performance on the benchmark dataset. Besides, our model is extensible to incorporate other function-related information to further improve the performance. Graph2GO does not rely on any manually crafted features and is entirely data-driven. Graph2GO is also applicable to other similar scenarios, such as predicting new therapeutic applications of existing drugs, since the learned embeddings can be used as feature inputs for various downstream machine learning tasks.

Methods

Graph2GO consists of two parts: the first part is an unsupervised graph-based representation model that utilizes both network information (protein-protein interactions, sequence similarities) and node attributes (protein sequence, subcellular location and protein domains) to generate unique embedding vectors for each protein; the second part is a fully-connected deep neural network classifier which use embeddings as features and Gene Ontology (GO) [27] as function labels. Gene Ontology (GO) defines concepts that describe functions and classifies functions on three aspects: molecular function (MF), cellular component (CC) and biological process (BP) [27]. We first describe how we obtain and encode different kinds of information, and then describe the detailed model specification. In Figure 1, we use PTEN gene as an example to detail how we encode these information. The model architecture is shown in Figure 2.

Dataset

We use reviewed and manually annotated proteins from SwissProt (release 2018_11) [15]. The dataset contains 20412 human proteins. The dataset provides GO annotations along with the experimental evidence code. We also obtained protein sequence, subcellular location and protein domains (Pfam) from SwissProt. We downloaded protein-protein interaction networks from STRING database (v10.5) [16], filtered by the proteins we obtained from SwissProt.

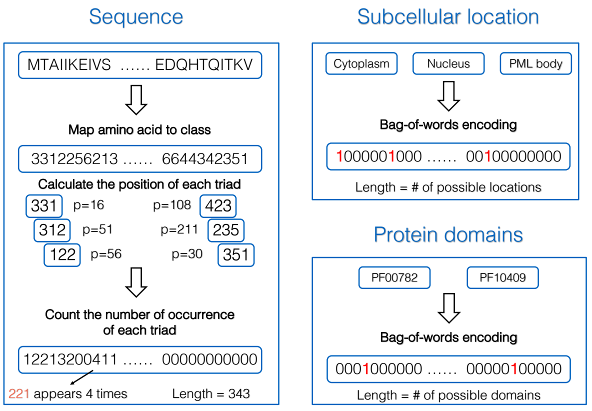

Figure 1. Here we use PTEN as an example to show how we encode sequence, subcellular location and protein domains features. For sequence encoding, we use the conjoint triad (CT) method. For subcellular location and protein domains, we use bag-of-words encoding.

Data encoding

Protein-protein interaction network

From the network data in STRING, we use the "combined score" provided by STRING as the confidence score. As shown in Figure S2, We perform the cross-validation to choose the threshold of the combined score for building the PPI network, and we only use interactions whose "combined score" are greater than 300 to construct the adjacency matrix as the representation of the network. For interactions with low confidence and protein pairs with no interactions mentioned in STRING, we assign the corresponding element in the adjacency matrix as zero.

Sequence similarity network

In order to build a sequence similarity network, we use the BLAST program to search similar sequences for each protein in our dataset [28]. We only select similarity pairs whose "e-value" are smaller than 1e-4 as candidate edges for similarity network. Detailed cross-validation results on selecting the threshold are shown in Figure S1.

Protein sequence

We encode amino acid sequences following the conjoint triad (CT) method [29], which has been widely used to represent sequence in related fields [30, 31, 32, 33]. The twenty kinds of amino acids are first clustered into seven classes according to the dipoles and volumes of the side chains since amino acids within the same class likely involve synonymous mutations. And then all the amino acids in the same class are considered as identical. The mapping between classes and amino acids is shown in Table S1. Then we consider any three continuous amino acids as a unit and counts the triad frequencies by calculating the occurrence numbers within the protein sequence. Thus, the dimension of the CT encoding is $7 \times 7 \times 7 = 343$. Using the CT method we manage to convert amino acid sequences into fixed-dimension representation.

Subcellular location

We obtained subcellular location information from SwissProt dataset. There are in total 359 different subcellular locations. We use bag-of-words encoding to represent this information, that's to say, the location is encoded as a binary vector of length 359 with each element indicating whether the protein is annotated with this location. Therefore, for a protein without any subcellular location annotation, it is represented as a vector of all zeros.

Protein domains

In the dataset we obtained from SwissProt, there are in total 5817 unique protein domains. In order to avoid the curse of dimensionality and to decrease the complexity, we only use protein domain terms that appear more than 5 times in our dataset and we are left with 655 terms. The protein domain knowledge is also encoded by way of bag-of-words encoding.

VGAE model

In the first part of Graph2GO, the core is a Variational Graph Auto-encoder (VGAE) [34], which can generate latent representations based on both network structure and node features, as shown in Figure 2a. We will discuss this model by problem formulation, followed by its inference part (encoder) and generative part (decoder). The purpose of VGAE is to learn interpretable embedding for each protein by training the encoder and decoder at the same time.

Problem formulation

We are given an undirected graph $\mathcal{G} = (\mathcal{V}, \mathcal{E})$ with $N = |\mathcal{V}|$ nodes. Here N is the number of proteins, and each vertex of $\mathcal{G}$ represents one protein while each edge is one interaction in the PPI network or a similarity pair in the sequence similarity network. The adjacency matrix $\mathbf{A}$ of $\mathcal{G}$ and its degree matrix $\mathbf{D}_A$ are derived from known network information. We enforce self-loops in the graph by simply adding the identity matrix to $\mathbf{A}$. The input features of each vertex are included in an $N \times R$ matrix $\mathbf{X}$, which is the concatenation of protein sequence feature $\mathbf{S}$, subcellular location feature $\mathbf{L}$ and protein domains feature $\mathbf{D}$ as shown in Figure 2a. Here R is the sum of feature dimensions of matrix $\mathbf{S}$, $\mathbf{L}$ and $\mathbf{D}$.

The model depicted in Figure 2a is basically an encoder-decoder model. First the encoder maps each node v_i in the graph to a low-dimensional latent variable, $\mathbf{z}_i$, based on the node's position in the graph, its local neighborhood structure, and its attributes. Next, the decoder reconstructs the adjacency matrix term A_{ij} corresponding to the pair of nodes v_i and v_j. By jointly optimizing the encoder and decoder, the model learns to compress graph structure information and original node attributes into the low-dimensional latent space. In principle, the model learns to propagate features across the proteins based on the network structure and the encoder-decoder architecture ensures that the learned representations in the latent space are meaningful and informative.

Encoder

The inference module is a graph convolutional network (GCN) encoder [35], which is a function with the goal of a mapping from the original features $\mathbf{X}$ to the latent variable $\mathbf{Z}$ with the network information $\mathbf{A}$. To be more specific, we want to learn

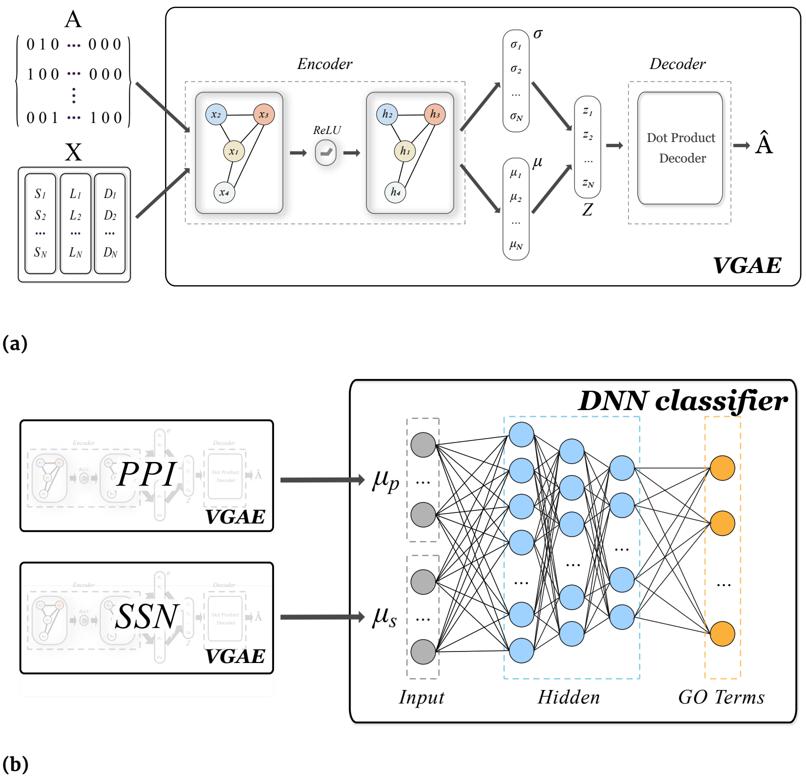

Figure 2. **(a)** Architecture of the variational graph auto encoder (VGAE), the first part of Graph2GO. The inputs to VGAE include an adjacency matrix A, representing protein-related network, and a node attribute matrix. VGAE is an encoder-decoder approach. The encoder is a two-layer graph convolutional networks and the decoder is a dot product decoder. The mean vector μ_i is the output embedding for classification. **(b)** Graph2GO pipeline consisting of two VGAE models for protein-protein interaction (PPI) networks and sequence similarity networks (SSN), respectively, and the final deep neural network (DNN) classifier. Two embeddings generated from PPI network and SSN are concatenated as the input for DNN classifier, and the DNN classifier outputs the probabilities of the protein having each GO term annotation.

a probability model $q(\mathbf{Z}|\mathbf{X}, \mathbf{A})$ and here we use graph convolutional network g to model this probability:

$$[\mu; \log \sigma] = g(\mathbf{X}, \mathbf{A}; \phi) \quad (1)$$

$$q(\mathbf{Z}|\mathbf{X}, \mathbf{A}) = \mathcal{N}(\mathbf{Z}; \mu, \sigma^2 \mathbf{I}) \quad (2)$$

where q is a function that encodes proteins into latent variables $\mathbf{Z}$ based on network information $\mathbf{A}$ and node attributes $\mathbf{X}$, ϕ is the parameter of graph convolutional network g and $\mathbf{I}$ is an identity matrix. μ and σ are mean and variance of the Gaussian distribution corresponding to latent variable $\mathbf{Z}$ that are estimated using network g from data directly. Then $\mathbf{Z}$ can be sampled from $q(\mathbf{Z}|\mathbf{X}, \mathbf{A})$. According to the reparameterization trick [36], z_i is obtained by:

$$\mathbf{z}_i = \mu + \sigma \odot \epsilon_i \quad (3)$$

where $\odot$ is element-wise multiplication and $\epsilon_i \sim \mathcal{N}(0, \mathbf{I})$.

Our graph convolutional network g is a two-layer network as defined:

$$g(\mathbf{X}, \mathbf{A}; \phi) = \tilde{A}\,\mathrm{ReLU}(\tilde{A}XW^{(0)})\mathbf{W}^{(1)} \quad (4)$$

where $\mathbf{W}^{(i)}$ are parameter matrices we need to train, $\mathrm{ReLU}(\cdot) = max(0, \cdot)$ is the activation function and $\tilde{A} = \mathbf{D}_A^{-\frac{1}{2}}\mathbf{A}\mathbf{D}_A^{-\frac{1}{2}}$ is the symmetrically normalized adjacency matrix [35].

The intuition of GCN is as follows. The multiplication of feature matrix $\mathbf{X}$ and the adjacency matrix $\mathbf{A}$ means that, for every node, we sum up feature vectors of all its neighboring nodes and itself to update its feature representation. In this way, node attributes are propagated across the graph and the information associated with each protein is augmented, especially for proteins with little annotation. This graph convolution operation is similar to Laplacian smoothing which makes features of nodes in the same cluster similar [37]. In order to avoid changing the scale of the feature vectors, $\mathbf{A}$ is first normalized so that all rows sum to one by symmetric normalization. Multiplying $\mathbf{X}$ with $\tilde{\mathbf{A}}$ means taking the average of neighboring node's features. In this way, GCN can effectively learn embeddings through integrating neighboring graph features.

Decoder

As our latent embedding already contains both node attributes and structure information, the generative module we define here is a simple inner product decoder that aims to reconstruct adjacency matrix $\mathbf{A}$ using learned latent variables $\mathbf{z}_i$:

$$p(\mathbf{A}|\mathbf{Z}) = \prod_{i=1}^{N}\prod_{j=1}^{N} p(A_{ij}|\mathbf{z}_i, \mathbf{z}_j) \quad (5)$$

$$p(A_{ij} = 1|\mathbf{z}_i, \mathbf{z}_j) = \sigma(\mathbf{z}_i^\top \mathbf{z}_j) \quad (6)$$

where $\sigma(\cdot)$ is the logistic function. We use the logistic function transformed inner product of $\mathbf{z}_i$ and $\mathbf{z}_j$ (right-hand side of Equ. (6)) as the probability of these two proteins having interaction. As indicated in Figure 2a, the output of the decoder $\hat{\mathbf{A}}$ is the approximation of adjacency matrix $\mathbf{A}$ and we optimize the model so as to make them as close as possible.

Cost function

Similar to VAE [36], the cost function is the reconstruction error with a regularizer:

$$\mathcal{L} = \mathbb{E}_{q(\mathbf{Z}|\mathbf{X},\mathbf{A})}[\log p(\mathbf{A}|\mathbf{Z})] - \mathrm{KL}[q(\mathbf{Z}|\mathbf{X},\mathbf{A})||p(\mathbf{Z})] \tag{7}$$

where $KL[q(\cdot)||p(\cdot)]$ is the Kullback-Leibler divergence between $q(\cdot)$ and $p(\cdot)$. The first term is to minimize the reconstruction error of the adjacency matrix $\mathbf{A}$. The second term is to minimize the difference between $q(\mathbf{Z}|\mathbf{X},\mathbf{A})$ and $p(\mathbf{Z})$. The cost function is the tradeoff between how accurate our model can reconstruct the input network and how close the latent variables can match $p(\mathbf{Z})$. As specified in VAE, we assume $p(\mathbf{Z}) \sim \mathcal{N}(0,\mathbf{I})$. We train the VGAE using stochastic gradient descent to optimize the cost function with respect to the parameters of the encoder [36].

Deep neural network classifier

In the second part of Graph2GO, as shown in Figure 2b, we take out embeddings μ_i contained in the matrix μ and train a fully-connected deep neural network as the final supervised classifier. Deep neural network (DNN) has become one of the most popular and powerful techniques for supervised machine learning [38]. It consists of three types of layers: input layer, hidden layers and output layer. The inputs to the classifier are embedding vectors for each protein, while the output layer represents GO terms that we aim to predict. Hidden layers are stacked between the input layer and output layer, aiming to learn meaningful abstract features for the task. To go from one layer to the next, a set of units (neurons) compute a weighted sum of their inputs from the previous layer and pass the result through a non-linear function (activation function). By stacking multiple hidden layers, DNN can learn an extremely intricate non-linear function mapping from the input to the output, which means it works best when the task is inherently non-linear. Gradient descent algorithm is commonly used for training neural networks and binary cross-entropy is used as the cost function in our multi-label classification task.

We train three classifiers for molecular function (MF), biological processes (BP) and cellular components (CC), respectively. For each classifier, it is a multi-class multi-label model and the dimension of the output space is the number of GO terms within each ontology. Each protein may be predicted with multiple GO terms simultaneously. The classifier predicts the probabilities of the protein having each GO term annotation. The performance of the classifier can be excellent without much complex neural network structures since the embeddings already contain enough information and are highly representative in the learned low-dimensional vector space.

Graph2GO pipeline

As shown in Figure 2b, Graph2GO pipeline consists of two VGAE models for the protein-protein interaction (PPI) network and sequence similarity network (SSN), respectively, and the final deep neural network (DNN) classifier for predicting protein functions. Instead of combining two networks first and train one VGAE to obtain overall embeddings, by training independent VGAE model for each network and combine their embeddings, we try to avoid introducing noise and to keep as much information as possible. We compared the performance of these two ways for integrating two networks and the detailed results is shown in Table S2.

Results

Experimental setup

Graph2GO was implemented using Tensorflow in Python and took advantage of the powerful computing capacity of GPU. All the simulations were carried out on Owens cluster provided by the Ohio Supercomputer Center (OSC) [39] with 27 processors and 127GB memory. The GPU we used was NVIDIA Tesla P100 with 16GB memory. Our source code is available at https://github.com/yanzhanglab/Graph2GO.

For our experiments, we use SwissProt's reviewed and manually annotated human proteins (release 2018_11). We only use proteins that also exist in the STRING database (v10.5) where corresponding interaction information is available. In order to use the CT method to encode amino acid sequences, we delete proteins whose sequence contains ambiguous amino acids including B, O, J, U, X and Z. After filtering out 5279 proteins, our final dataset contains 15133 proteins, along with 1,713,652 protein-protein interactions and 843,212 edges in the sequence similarity network. For GO annotations, we only consider the experimental evidence code among EXP, IDA, IPI, IMP, IGI, IEP, TAS and IC [27]. If a protein is annotated with a GO term, we additionally annotated it with all the ancestor terms.

In the first part of our architecture, the encoder is a two-layer neural network structure of 400 and 800 neurons each. Using two layers of graph convolutional operations is recommended in [35, 37], and we confirm the choice of two layers by performing the cross-validation, as shown in Figure S1. We initialize weights as described in [40] and train the model for 200 iterations using Adam algorithm with a learning rate of 0.001 [41]. The final prediction classifier is a three-layer fully-connected neural network with 1024, 512 and 256 neurons, respectively. Dropout layer and batch normalization layer are used between every dense layers to avoid over-fitting and the dropout rate is set as 0.3. Adam is used to train the model for 100 iterations with a learning rate of 0.001.

Evaluation metric

We use metrics that are similar to those adopted by the CAFA (Critical Assessment of protein Function Annotation algorithms) challenge to evaluate our model and compare with others [1]. The first metric we use is the maximum F-measure over all possible thresholds defined as follows:

$$pr(t) = \frac{\sum_i \sum_f I(f \in P_i(t) \wedge f \in T_i)}{\sum_i \sum_f I(f \in P_i(t))} \tag{8}$$

$$rc(t) = \frac{\sum_i \sum_f I(f \in P_i(t) \wedge f \in T_i)}{\sum_i \sum_f I(f \in T_i)} \tag{9}$$

$$F_{\max} = \max_t \left\{ \frac{2 \cdot pr(t) \cdot rc(t)}{pr(t) + rc(t)} \right\} \tag{10}$$

where pr means precision, rc means recall, I is the indicator function, f is a GO term, $P_i(t)$ is a set of predicted GO terms for protein i using the threshold t, and T_i is a set of annotated GO terms for protein i.

We also use similar term-centric metrics to evaluate the model as suggested by CAFA. However, instead of using receiver operating characteristic (ROC) curves, we choose to consider precision-recall (PR) curve and calculate the area under the curve. As pointed by Davis and Goadrich [42], when dealing with highly imbalanced datasets, precision-recall curves give a more informative picture of an algorithm's performance than ROC curves. Since this is a multi-label task, we adopt two averaged measures of area under precision-recall curve (AUPR) for

all terms:

$$pr_f(t) = \frac{\sum_i I(f \in P_i(t) \wedge f \in T_i)}{\sum_i I(f \in P_i(t))} \quad (11)$$

$$rc_f(t) = \frac{\sum_i I(f \in P_i(t) \wedge f \in T_i)}{\sum_i I(f \in T_i)} \quad (12)$$

$$\text{AUPR}_f = \sum_t (rc_f(t) - rc_f(t-1)) \cdot pr_f(t) \quad (13)$$

$$\text{M-AUPR} = \frac{1}{N_f} \cdot \sum_f \text{AUPR}_f \quad (14)$$

$$\text{m-AUPR} = \sum_t (rc(t) - rc(t-1)) \cdot pr(t) \quad (15)$$

Where pr_f and rc_f are precision and recall for a single GO term f, AUPR_f is the area under the precision-recall curve (AUPR) for f, N_f is the number of GO terms used for evaluation. Macro-averaged AUPR (M-AUPR) is defined as the unweighted mean of the AUPR for all labels, while micro-averaged AUPR (m-AUPR) is calculated globally by considering each element of the label indicator matrix as a label.

Comparison between different types of features

In this section, we compare the results of using different network information and node attributes. The purpose is to show which type of feature is most informative and how we can further improve the performance by combining multiple types of features. In Table 1, we show the results when using different network types and node attributes. For network types, we test among only using PPI network, only sequence similarity network and the combined network. As for node attributes, we compare the performance among different attributes (sequence, protein domains and subcellular location), and especially, compare with ALL attributes (using all three kinds of attributes together). We do not consider sequence information as node attributes when the sequence similarity network is used as the network to train the model because of the redundancy.

Looking at the performance of using different network types, the combined network is always better than the other two single network types across all three ontologies, which indicates that the PPI network and the sequence similarity network can provide complementary information for function prediction. Comparing the effect of PPI network and sequence similarity network, PPI network shows better performance than sequence similarity network except on molecular function (MF). We can conclude that PPI network provides more function-related information than sequence similarity network and that MF ontology has a great correlation with sequence information.

From the analysis of the effects of using different node attributes, we can conclude that the integration of all node attributes is better than using any single of them across all three ontologies, no matter what kind of network type is used. We can see that using sequence attribute can achieve reasonable performance for predicting functions in all three ontologies when combined with PPI network, proving the importance of sequence information. It is also shown that protein domain attribute is the most informative feature for predicting MF functions while subcellular location attribute is important for CC functions' prediction. Overall, each type of node attribute contributes to the function prediction, and the combination of all features improves the performance and provides more robust predictions. Based on the results shown in Table 1, our final model considers both sequence similarity network and PPI network as our network information. As for node attributes, we include subcellular location and protein domain features as node attributes in the sequence similarity network while include all three types of features as node attributes in the PPI network.

Comparison with BLAST

We first compare our method with a baseline model, BLAST, which is a widely used homology-based method for annotating protein functions [5]. We randomly split the dataset into 80% training set and 20% test set. We compare our method with BLAST under two conditions: (1) full dataset: using all 80% training set; (2) partial dataset: removing training samples with more than 50% identity with one of the test samples (removing potential homologs). The training set is used for constructing the database. Then all GO term annotations of the best hit in the database are assigned to the query protein in the test set as the predicted functions. Since BLAST cannot assign probabilities for each prediction, we use Precision, Recall and F1-score as the evaluation metrics instead of the M-AUPR, m-AUPR and F-max we used in the previous section. For Graph2GO, we use the same training and test set and use 0.5 as the threshold to assign predicted labels.

As shown in Table 2, in terms of all metrics, Graph2GO outperforms BLAST by a large margin across all three ontologies under both comparison condition, especially for CC and BP, proving the superiority of our model. We can see that Graph2GO can achieve significantly higher Precision than

		CC			MF			BP		
Network	Attribute	M-AUPR	m-AUPR	F-max	M-AUPR	m-AUPR	F-max	M-AUPR	m-AUPR	F-max
sequence	location	0.325	0.686	0.639	0.411	0.650	0.624	0.152	0.336	0.388
	domain	0.217	0.594	0.568	0.383	0.669	0.639	0.144	0.357	0.384
	ALL	0.348	0.694	0.644	0.465	0.717	0.671	0.185	0.390	0.418
PPI	sequence	0.471	0.683	0.639	0.466	0.657	0.626	0.268	0.458	0.472
	location	0.448	0.704	0.652	0.398	0.596	0.567	0.233	0.435	0.450
	domain	0.443	0.681	0.634	0.476	0.678	0.635	0.258	0.464	0.470
	ALL	0.478	0.716	0.644	0.478	0.677	0.623	0.268	0.471	0.471
combined	location	0.465	0.744	0.686	0.499	0.715	0.672	0.239	0.448	0.463
	domain	0.426	0.695	0.643	0.520	0.751	0.698	0.253	0.465	0.473
	ALL	**0.494**	**0.751**	**0.686**	**0.560**	**0.761**	**0.718**	**0.284**	**0.488**	**0.490**

Table 1. Performance comparison among using different network types (sequence similarity network, PPI network and both) and node attributes (sequence, subcellular location, protein domains and ALL). We use the M-AUPR, m-AUPR and F-max as the evaluation metric. ALL means using the combination of all features as node attributes, and we do not include sequence as node attributes when sequence similarity network is used to train the model.

Method	CC Precision	CC Recall	CC F1-score	MF Precision	MF Recall	MF F1-score	BP Precision	BP Recall	BP F1-score
BLAST (full)	0.540	0.589	0.564	0.633	0.712	0.670	0.373	0.427	0.398
BLAST (partial)	0.396	0.429	0.412	0.421	0.490	0.453	0.234	0.271	0.251
Graph2GO (full)	0.766	0.624	0.688	0.816	0.639	0.717	0.686	0.354	0.467
Graph2GO (partial)	0.731	0.549	0.627	0.698	0.448	0.545	0.595	0.286	0.387

Table 2. Performance comparison between BLAST and Graph2GO in terms of precision, recall and f1-score under two conditions (full dataset and partial dataset after removing homologs). We use 0.5 as the threshold to assign predicted labels for Graph2GO. It should be noted that the F1 score here is different from the F-max used previously since the threshold is pre-specified for Graph2GO and there is no threshold for BLAST.

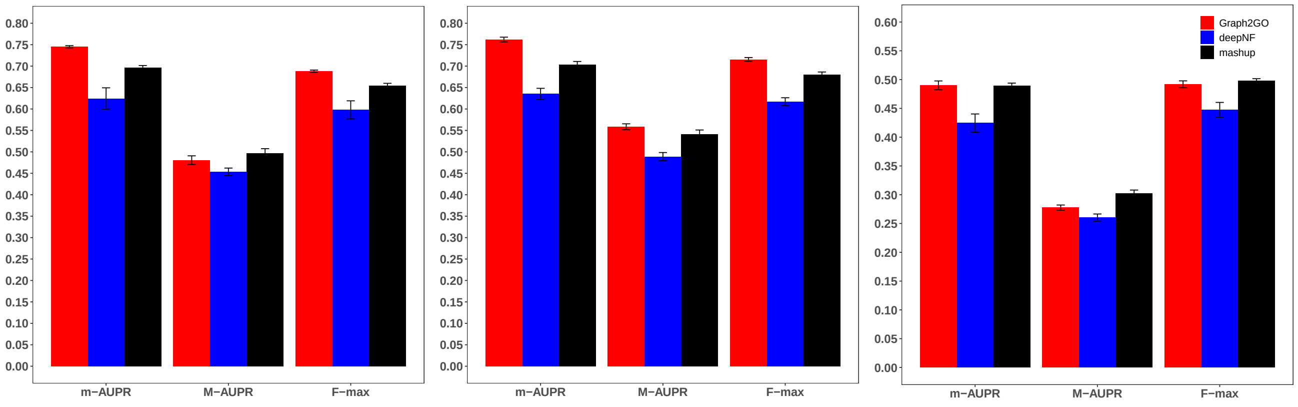

(a) CC **(b)** MF **(c)** BP

Figure 3. Performance comparison with other network-based methods. Graph2GO is compared with Mashup and deepNF in terms of three metrics: micro-AUPR, macro-AUPR and F-max. Figure (a) - (c) shows the comparison results on CC, MF and BP ontology, respectively. Each method is evaluated using 5-fold cross-validation, repeated 10 times to calculate the confidence interval.

BLAST under the full dataset condition. BLAST results in many false positives and low Precision because of predicting functions simply based on amino acid sequence information while ignoring other informative features. Besides, it is shown that BLAST can obtain a reasonable performance in MF ontology, which demonstrates that amino acid sequence information is highly related to the functions in MF ontology again. After removing highly similar sequences of the test set in the training set, performances of both methods drop accordingly, while Graph2GO shows a smaller drop and thus outperforms BLAST even more under the partial dataset condition. For BLAST, the performance drops from full dataset condition to partial dataset condition in terms of F1-score is 27%, 32% and 37% for CC, MF and BP, respectively. As for Graph2GO, the F1-score drops 9%, 25% and 17% accordingly, which are significantly less than BLAST, exhibiting a more robust performance.

Comparison with other network-based methods

To evaluate the performance of our method, we compare our method with two popular protein function prediction methods: Mashup and deepNF, which are also based on the concept of embedding. In their initial implementations, Mashup and deepNF use SVM as the classification model. In order to make it more comparable, we also use DNN that is used by Graph2GO as their classification models. Each method is evaluated using 5-fold cross-validation, repeated 10 times. We first compare the performance using all GO terms as shown in Figure 3. We also group GO terms into three functional categories based on the sparsity levels for each ontology, each containing GO terms with 11-30, 31-100 and 101-300 proteins, and show the detailed comparison in Figures S2, S3 and S4.

For both CC and MF ontology, our model outperforms deepNF and Mashup in terms of m-AUPR and F-max, and achieves comparable results with deepNF and Mashup in terms of M-AUPR. Our model is comparable to Mashup and outperforms deepNF in BP ontology in terms of m-AUPR and F-max and slightly worse than Mashup in M-AUPR. Looking at the detailed comparison in three sparsity levels shown in Figure S2, our model can achieve the best performance consistently for MF ontology, across all sparsity levels. As we discussed above, the MF ontology has a great correlation with sequence information. Unlike Mashup and deepNF that only considers PPI networks, Graph2GO explicitly includes sequence information in the model (sequence similarity network and sequence in the node attribute), which improves the performance of our model. As for CC ontology, Graph2GO can achieve the best performance for the most general categories (i.e., annotating between 101 and 300 proteins). For GO terms annotated between 11 and 100 proteins, our method is not as good as Mashup and deepNF. For BP ontology, as the number of annotations per GO term increases, the performance of Graph2GO gradually improves and become comparable with other two methods.

It can be observed that Graph2GO works best for GO terms with enough annotated proteins, and is not as good as Mashup for very specific GO terms with few annotations for CC and BP ontology. In summary, our model can achieve state-of-the-art performance in both CC and MF ontology, especially as the number of annotated proteins increases, and obtain comparable results in BP ontology. Compared with Mashup and deepNF, we can conclude that both the inclusion of discriminating node attributes and the power of attribute propagation across two types of networks enable Graph2GO to obtain competing results.

	CC			MF			BP		
Species	M-AUPR	m-AUPR	F-max	M-AUPR	m-AUPR	F-max	M-AUPR	m-AUPR	F-max
human	0.494	0.751	0.686	0.560	0.761	0.718	0.284	0.488	0.490
fruit fly	0.570	0.764	0.729	0.599	0.710	0.693	0.388	0.531	0.530
mouse	0.480	0.743	0.681	0.583	0.763	0.710	0.262	0.482	0.483
rat	0.461	0.701	0.652	0.577	0.743	0.699	0.267	0.429	0.448
s. cerevisiae	0.677	0.848	0.781	0.597	0.740	0.689	0.481	0.647	0.620
bacillus subtilis	0.709	0.860	0.803	0.620	0.742	0.693	0.577	0.665	0.651

Table 3. Performance on other five species in terms of macro-AUPR, micro-AUPR and F-max metrics.

Performance on other species

In order to demonstrate the power and robustness of Graph2GO, We downloaded data from SwissProt and STRING database for other five species (fruit fly, mouse, rat, *s. cerevisiae* and *bacillus subtilis*), and tested the performance of Graph2GO on these species. For each species, we followed the same procedure as we tested on human dataset, and the performance was evaluated in each species individually. Table 3 shows the results in terms of macro-AUPR, micro-AUPR and F-max. We observe that Graph2GO can achieve consistently decent performance on all five species in terms of these evaluation metrics, which proves the robustness of our method. It should be noted that the performance on *S. cerevisiae* and *Bacillus subtilis* is a lot better than on other species.

Graph2GO web server

We also developed a web server based on Shiny app in R that supports protein function query on-the-fly: https://integrativeomics.shinyapps.io/graph2go/. Currently, the web server supports 15133 Human proteins that are trained by our model. Network information (sequence similarity network and PPI network) and node attributes (subcellular location and protein domains) are displayed in our web server. The function prediction results are ranked by the probability score for each ontology separately, and users can specify the threshold to control the confidence of the results.

One of the promising applications of our model is to utilize the latent representations to perform downstream analysis, since the generated representations already summarize various kinds of informative features. We support downstream clustering analysis for the query protein where similarities are measured based on latent representations. Most similar proteins can be detected for the query protein to constitute a network module. Gene ontology enrichment analysis can be performed on all proteins in the network module to analyze functions of the module. In the future, we are going to extend the server to include more proteins from other species.

Discussion

Feature transfer for solving annotation bias

One of the challenges in protein function prediction is the lack of annotated features for proteins, which are crucial for machine learning models. This is due to the bias in the experimental annotation of proteins and has impeded the development of biomedical research to some extent [43, 44]. In our experiment on the human dataset, among 15133 proteins, 5716 proteins lack annotation of protein domains while 2364 lack annotation of subcellular location. Even with lots of missing values, when only using protein domains or subcellular location as node attributes, Graph2GO can still achieve reasonable results, as shown in Table 1. To demonstrate the power of Graph2GO to predict on these sparsely annotated proteins, we divide the test set into two groups: the sparsity group (without any subcellular location or protein domain annotation) and the non-sparsity group. We compare Graph2GO with a convolutional neural network (CNN) model. This CNN model uses the same node attributes as Graph2GO while do not utilize networks to propagate protein attributes on these two groups separately. The detailed description of this CNN model is in the supplementary material. The comparison results are shown in Table S3. It is anticipated t hat the performance on sparsity group should be worse than that of non-sparsity group due to the lack of features. We calculate the ratio between the performance of sparsity group and non-sparsity to measure how well Graph2GO and CNN handle the lack of features. The averaged ratio in terms of F-max for CNN is 74%, 44% and 73% for CC, MF and BP, respectively, while for Graph2GO, the averaged ratio is 101%, 84% and 111%. We can see that when lacking features, the performance of CNN would drop significantly compared to the non-sparsity group. However, the performance of Graph2GO is much more stable when facing the lack of features. In some cases, the performance on sparsity group is even better than non-sparsity group. This is due to the feature transfer of Graph2GO where node attributes are transferred between nodes in the graph to augment the information of each protein. Since proteins are connected in the interaction network, nodes without any attributes can still update their representations based on attributes of neighboring nodes, which solves the problem of missing values to some extent.

Encoding of original features into the model is of high importance to Graph2GO, not only for preserving information as much as possible, but also for feature transfer. We represent protein domains and subcellular location using bag-of-words encoding method, which best preserves the original information and supports the addition of features that the feature transfer relies on. As for protein sequence, CT encoding method loses sequential information and is not well suited for the addition operation. To solve this, we also utilize the similarity network to represent the sequence information, and we discover that the inclusion of both types of sequence representations is better than any single one of them, which might indicate that they can provide some complementary information.

Extensibility and future work

Compared to other methods that also use multiple protein features [17, 13, 14], one of the advantages of our method is that it is convenient to incorporate other function-related information. Within the network architecture, all the features are regarded as node attributes and can be treated equally. In this paper, we found the integration of all these node features and the network information provides the best prediction performance. The model can be easily extended to take into account additional information such as post-translational modifications, protein structure or any other protein features. It's also worth

incorporating more networks and find a better way to integrate them. For example, we may use the seven channels in STRING database separtely instead of using the combined PPI network, as adopted by Mashup.

The architecture we proposed in this paper can not only be used to predict protein functions, but also be applied to other tasks which involve biological networks, since the learned embeddings are informative and can be used as feature inputs for various downstream machine learning tasks. For example, one can predict the interactions between any two proteins, predict new therapeutic applications of existing drug or run clustering algorithm to cluster genes into modules based on learned embeddings.

In principle, predicting GO terms is a hierarchical classification problem, since GO terms are organized in a hierarchy and are interacting with each other [45]. Currently, Graph2GO assumes the independence of all GO terms, which might cause inconsistence between the prediction of leaf GO terms and the corresponding parent GO terms. In the future, we will consider adding some constraints on the output layer of our classification model to force the prediction consistent between leaf nodes and parent nodes. We will also try to incorporate the GO hierarchy into the model as one of the network structures to inform the training process.

Conclusion

In this work, we present Graph2GO, a graph-based deep learning model that can make use of heterogeneous information in a unified way to predict protein functions. Our network representation learning method can take into account both network structure and protein attributes together to make predictions by organizing proteins into an attributed network architecture. Graph2GO achieves state-of-the-art performance on the benchmark dataset and is easy to be adapted to solve other tasks involving biological networks, such as link prediction, node classification and sub-network discovery.

Availability of source code and requirements (optional, if code is present)

- Project name: Graph2GO
- Project home page: https://github.com/yanzhanglab/Graph2GO
- Operating system(s): Platform independent
- Programming language: Python, R
- Other requirements: not applicable
- License: MIT

Availability of supporting data and materials

All data is publicly available in our Github project homepage. All the supplementary files are available on GigaDB upon publication.

Additional files

There is an additional Supplementary Materials file containing additional information. It includes the following figures and tables:

Supplementary Figure 1. Cross-validation results of choosing different number of layers for GCN model. The performances on all three ontologies reach the best when using two layers of graph convolutional operators. As the number of layers becomes larger, the performance gets worse because applying graph convolutional operator repeatedly may mix of node features from different clusters and make them indistinguishable.

Supplementary Figure 2. Performance comparison with other state-of-the-art methods in CC ontology for three different sparsity levels. Graph2GO is compared with Mashup and deepNF in terms of three metrics: micro-AUPR, macro-AUPR and F-max. There three figures shows the results of three sparsity levels: [11-30], [31-100] and [101-300]. Each method is evaluated using 5-fold cross-validation, repeated 10 times to calculate the confidence interval.

Supplementary Figure 3. Performance comparison with other state-of-the-art methods in MF ontology for three different sparsity levels. Graph2GO is compared with Mashup and deepNF in terms of three metrics: micro-AUPR, macro-AUPR and F-max. There three figures shows the results of three sparsity levels: [11-30], [31-100] and [101-300]. Each method is evaluated using 5-fold cross-validation, repeated 10 times to calculate the confidence interval.

Supplementary Figure 4. Performance comparison with other state-of-the-art methods in BP ontology for three different sparsity levels. Graph2GO is compared with Mashup and deepNF in terms of three metrics: micro-AUPR, macro-AUPR and F-max. There three figures shows the results of three sparsity levels: [11-30], [31-100] and [101-300]. Each method is evaluated using 5-fold cross-validation, repeated 10 times to calculate the confidence interval.

Supplementary Table 1. Classification of amino acids according to their dipoles and volumes of the side chains based on the CT method.

Supplementary Table 2. Performance between the model where we train independent VGAE for each network and combine their embeddings (refers as "individual") and where we first combine the networks and train one VGAE to obtain the overall embedding (refers as "combined") in terms of M-AUPR, m-AUPR and F-max. The experiment procedure is the same as other experiments performed in the "Results" section and is detailed described there.

Supplementary Table 3. Performance of Graph2GO and CNN on sparsity group and non-sparsity group of the test set in terms of F1-score. Here the "sp" means the sparsity group while "non" means non-sparsity group.

Declarations

List of abbreviations

BLAST: Basic Local Alignment and Search Tool; BP: biological process; CC: cellular component; CNN: convolutional neural network; CT: conjoint triad; DNN: deep neural network; GCN: graph convolutional network; GO: Gene Ontology; GPU: graphics processing unit; MF: molecular function; M-AUPR: macro-averaged area under the precision-recall curve; m-AUPR: micro-averaged area under the precision-recall curve; OSC: Ohio Supercomputer Center; PPI: protein-protein interaction; ROC: receiver operating characteristic; SSN: sequence similarity network; SVM: support vector machine

Consent for publication

Not applicable

Competing Interests

Y.G. serves as a consultant at Eli Lilly and Company and a consultant at Merck Group.

Funding

This work was supported by The Ohio State University Startup Funds to Y. Z. and aslo partially supported by the NCI OSU Comprehensive Cancer Center Support Grant [P30CA016058, PI: Pollock] to Y. Z.

Author's Contributions

YZ and YG conceived the project. KF performed the analysis and developed the tools. YZ and YG supervised the study. KF and YZ drafted the manuscript. All authors read and approved the final manuscript.

Acknowledgements

We acknowledge the use of the compute resources of Ohio Supercomputer Center (OSC).

References

1. Radivojac P, Clark WT, Oron TR, Schnoes AM, Wittkop T, Sokolov A, et al. A large-scale evaluation of computational protein function prediction. Nature methods 2013;10(3):221.
2. Loewenstein Y, Raimondo D, Redfern OC, Watson J, Frishman D, Linial M, et al. Protein function annotation by homology-based inference. Genome biology 2009;10(2):207.
3. Piovesan D, Luigi Martelli P, Fariselli P, Zauli A, Rossi I, Casadio R. BAR-PLUS: the Bologna Annotation Resource Plus for functional and structural annotation of protein sequences. Nucleic acids research 2011;39(suppl_2):W197–W202.
4. Chitale M, Hawkins T, Park C, Kihara D. ESG: extended similarity group method for automated protein function prediction. Bioinformatics 2009;25(14):1739–1745.
5. Jones CE, Baumann U, Brown AL. Automated methods of predicting the function of biological sequences using GO and BLAST. BMC bioinformatics 2005;6(1):272.
6. Sharan R, Ulitsky I, Shamir R. Network-based prediction of protein function. Molecular systems biology 2007;3(1):88.
7. Chua HN, Sung WK, Wong L. Using indirect protein interactions for the prediction of Gene Ontology functions. In: BMC bioinformatics, vol. 8 BioMed Central; 2007. p. S8.
8. Forslund K, Sonnhammer EL. Predicting protein function from domain content. Bioinformatics 2008;24(15):1681–1687.
9. Rentzsch R, Orengo CA. Protein function prediction using domain families. In: Bmc Bioinformatics, vol. 14 BioMed Central; 2013. p. S5.
10. Lee D, Redfern O, Orengo C. Predicting protein function from sequence and structure. Nature reviews molecular cell biology 2007;8(12):995.
11. Jensen LJ, Gupta R, Blom N, Devos D, Tamames J, Kesmir C, et al. Prediction of human protein function from post-translational modifications and localization features. Journal of molecular biology 2002;319(5):1257–1265.
12. Verspoor KM. Roles for text mining in protein function prediction. In: Biomedical Literature Mining Springer; 2014.p. 95–108.
13. Piovesan D, Giollo M, Leonardi E, Ferrari C, Tosatto SC. INGA: protein function prediction combining interaction networks, domain assignments and sequence similarity. Nucleic acids research 2015;43(W1):W134–W140.
14. Zhang C, Freddolino PL, Zhang Y. COFACTOR: improved protein function prediction by combining structure, sequence and protein–protein interaction information. Nucleic acids research 2017;45(W1):W291–W299.
15. Boutet E, Lieberherr D, Tognolli M, Schneider M, Bansal P, Bridge AJ, et al. UniProtKB/Swiss-Prot, the manually annotated section of the UniProt KnowledgeBase: how to use the entry view. In: Plant Bioinformatics Springer; 2016.p. 23–54.
16. Szklarczyk D, Morris JH, Cook H, Kuhn M, Wyder S, Simonovic M, et al. The STRING database in 2017: quality-controlled protein–protein association networks, made broadly accessible. Nucleic acids research 2016;p. gkw937.
17. Kulmanov M, Khan MA, Hoehndorf R. DeepGO: predicting protein functions from sequence and interactions using a deep ontology-aware classifier. Bioinformatics 2017;34(4):660–668.
18. Kearnes S, McCloskey K, Berndl M, Pande V, Riley P. Molecular graph convolutions: moving beyond fingerprints. Journal of computer-aided molecular design 2016;30(8):595–608.
19. Backstrom L, Leskovec J. Supervised random walks: predicting and recommending links in social networks. In: Proceedings of the fourth ACM international conference on Web search and data mining ACM; 2011. p. 635–644.
20. Duvenaud DK, Maclaurin D, Iparraguirre J, Bombarell R, Hirzel T, Aspuru-Guzik A, et al. Convolutional networks on graphs for learning molecular fingerprints. In: Advances in neural information processing systems; 2015. p. 2224–2232.
21. Hamilton W, Ying Z, Leskovec J. Inductive representation learning on large graphs. In: Advances in Neural Information Processing Systems; 2017. p. 1024–1034.
22. Cho H, Berger B, Peng J. Compact integration of multi-network topology for functional analysis of genes. Cell systems 2016;3(6):540–548.
23. Gligorijević V, Barot M, Bonneau R. deepNF: Deep network fusion for protein function prediction. Bioinformatics 2018;34(22):3873–3881.
24. Bhagat S, Cormode G, Muthukrishnan S. Node classification in social networks. In: Social network data analytics Springer; 2011.p. 115–148.
25. Vishwanathan SVN, Schraudolph NN, Kondor R, Borgwardt KM. Graph kernels. Journal of Machine Learning Research 2010;11(Apr):1201–1242.
26. Hamilton WL, Ying R, Leskovec J. Representation learning on graphs: Methods and applications. arXiv preprint arXiv:170905584 2017;.
27. Ashburner M, Ball CA, Blake JA, Botstein D, Butler H, Cherry JM, et al. Gene ontology: tool for the unification of biology. Nature genetics 2000;25(1):25.
28. Altschul SF, Madden TL, Schäffer AA, Zhang J, Zhang Z, Miller W, et al. Gapped BLAST and PSI-BLAST: a new generation of protein database search programs. Nucleic acids research 1997;25(17):3389–3402.
29. Shen J, Zhang J, Luo X, Zhu W, Yu K, Chen K, et al. Predicting protein–protein interactions based only on sequences information. Proceedings of the National Academy of Sciences 2007;104(11):4337–4341.
30. Muppirala UK, Honavar VG, Dobbs D. Predicting RNA-protein interactions using only sequence information. BMC bioinformatics 2011;12(1):489.

31. You ZH, Lei YK, Zhu L, Xia J, Wang B. Prediction of protein-protein interactions from amino acid sequences with ensemble extreme learning machines and principal component analysis. In: BMC bioinformatics, vol. 14 BioMed Central; 2013. p. S10.
32. Wang H, Hu X. Accurate prediction of nuclear receptors with conjoint triad feature. BMC bioinformatics 2015;16(1):402.
33. Wang J, Zhang L, Jia L, Ren Y, Yu G. Protein-protein interactions prediction using a novel local conjoint triad descriptor of amino acid sequences. International journal of molecular sciences 2017;18(11):2373.
34. Kipf TN, Welling M. Variational graph auto-encoders. arXiv preprint arXiv:161107308 2016;.
35. Kipf TN, Welling M. Semi-supervised classification with graph convolutional networks. arXiv preprint arXiv:160902907 2016;.
36. Kingma DP, Welling M. Auto-encoding variational bayes. arXiv preprint arXiv:13126114 2013;.
37. Li Q, Han Z, Wu XM. Deeper insights into graph convolutional networks for semi-supervised learning. In: Thirty-Second AAAI Conference on Artificial Intelligence; 2018. .
38. LeCun Y, Bengio Y, Hinton G. Deep learning. nature 2015;521(7553):436–444.
39. Center OS, Ohio Supercomputer Center; 1987. http://osc.edu/ark:/19495/f5s1ph73.
40. Glorot X, Bengio Y. Understanding the difficulty of training deep feedforward neural networks. In: Proceedings of the thirteenth international conference on artificial intelligence and statistics; 2010. p. 249–256.
41. Kingma DP, Ba J. Adam: A method for stochastic optimization. arXiv preprint arXiv:14126980 2014;.
42. Davis J, Goadrich M. The relationship between Precision-Recall and ROC curves. In: Proceedings of the 23rd international conference on Machine learning ACM; 2006. p. 233–240.
43. Haynes WA, Tomczak A, Khatri P. Gene annotation bias impedes biomedical research. Scientific reports 2018;8(1):1362.
44. Schnoes AM, Ream DC, Thorman AW, Babbitt PC, Friedberg I. Biases in the experimental annotations of protein function and their effect on our understanding of protein function space. PLoS computational biology 2013;9(5):e1003063.
45. Kahanda I, Funk CS, Ullah F, Verspoor KM, Ben-Hur A. A close look at protein function prediction evaluation protocols. GigaScience 2015;4(1):s13742-015.

Click here to access/download
Supplementary Material
Supplementary_materials_Final_20200430.pdf

Personal Cover
Click here to access/download;Personal Cover;YanZhang_CoverLetter_20200430_signed.pdf

THE OHIO STATE UNIVERSITY

Yan Zhang, Ph.D.
Assistant Professor
Department of Biomedical Informatics
College of Medicine
The Ohio State University
310-B Lincoln Tower, 1800 Cannon Drive
Columbus, OH 43210
Phone: (614) 688-9643
Email: Yan.Zhang@osumc.edu

April 30, 2020

Dear Editor,

We would like to submit our revision of the manuscript "Graph2GO: a multi-modal attributed network embedding method for inferring protein functions". We have thoroughly revised our manuscript and addressed all the points raised by the reviewers.

We thank you and the reviewers for all the great suggestions that have helped us make the project better. And we hope that this improved version will meet the journal's publication requirement. We look forward to your feedback.

Sincerely,

Yan Zhang

Yan Zhang, Ph.D.
Assistant Professor
Department of Biomedical Informatics
The Ohio State University

Manuscript

Click here to access/download;Personal Cover;Response letter_Final_20200430.pdf

Response letter

We thank the editors and reviewers for their insightful comments and suggestions and believe that after addressing each comment the manuscript is stronger. We have highlighted all our answers in red color.

Editor's comments:
Both reviewers point out several issues with the evaluation and comparison procedures, which may have introduced bias. Before making a decision on a revised manuscript we will seek further advice from our reviewers and I urge you to make an effort to fully address their comments.

Authors' answer: We appreciate the feedback. We have worked carefully to solve the problem concerning evaluation and comparison procedures that might introduce bias. We have added a new evaluation metric F-max that is widely used by the community to replace our previous F1-score that is only based on top three predictions to make the evaluation more reliable. We include the detailed definition of these evaluation metrics so that general readers can understand. We also elaborate more on our DNN classification model and the models we compare with as suggested by reviewers. For experiments, we have added more details to clarify any confusions. Besides, we make changes to get rid of any variations that might impede the comparability of the results. We also followed other suggestions carefully and explained how we addressed them in the following point-to-point response.

Reviewer #1's comments:

This work describes a new method that uses attributed network representation that uses both interaction networks and protein features to predict protein function, when the function are represented as GO terms. This method of integrating heterogeneous information is, to my knowledge, novel and interesting. The methodology and results are mostly well described (but see comments below).

Major comments:
My main concern is the rather low F1 scores when compared with the apparent better performance of the AUPR scores, as shown in Table 1. (Incidentally, of showing F1 scores, why not show average precision and recall so that the reader can establish the contribution of the precision and recall to the F1 score?) The F1 scores are quite low (0.29 for CC and MF, and 0.07 for BP). I believe the authors could explain that, especially in light of the overall good performance in Table 2. Again, Figure 3 shows a low performance of F1 (0.25-0.35) of F1. How does that work with the F1 of 0.717 reported in Table 2?

Authors' answer: Thanks for pointing out this problem. We should apologize for the inconsistence of the use of F1-score. In Table 1, Table 3 and Figure 3, the F1-score we used is only based on top three predictions, as used by Mashup and deepNF that we

want to compare with. In Table 2, when comparing with BLAST, we used the common F1-score, since F1-score based on top three predictions cannot be calculated for BLAST. The F1-score based on top three predictions tend to be small across all models, compared to other metrics. In order to solve the inconsistence, we decide to change the evaluation metric to F-max, the maximum F1-score over all possible thresholds. This evaluation metric is also adopted by CAFA (Critical Assessment of protein Function Annotation algorithms). The detailed definition of this metric along with others are introduced in section “Evaluation metric” under “Results”. Looking at the results based on F-max, the performance is more consistent with other metrics, and our model is still better than other methods under this metric.

I could not find definitions for m-AUPR and M-AUPR. It is my understanding that micro-averaged area under the precision-recall curve (m-AUPR) is computed by first vectorizing and then computing the AUPR; Macro-AUPR (M-AUPR) is computed by first computing the AUPR for each function separately, and then averaging these values across all functions. I would like to know how this was implemented here, and how the differences in performance are explained. While these performance metrics may be well-understood in the machine learning community, I think that the authors should elaborate a bit more on the choice of these metrics and their meaning, as Gigascience aims at a broader audience than those well-versed in machine learning.

Authors’ answer: Thanks for pointing out this issue. We have added a new section “Evaluation metric” under “Results” to elaborate on the metrics we used. Since the protein function prediction is a multi-label task, we need to evaluate the performance on all labels (GO terms). One of the most popular metrics used in this setting is macro- or micro- area under receiver operating characteristic (ROC) curves. However, given the fact that the dataset is kind of skewed (more negative samples than positive samples), the precision-recall curve will give a more informative picture of an algorithm’s performance than ROC curves, as pointed by Davis and Goadrich (reference 42 in the manuscript). Therefore, we adopt micro-AUPR (m-AUPR) and macro-AUPR (M-AUPR) as two evaluation metrics. m-AUPR is computed by first vectorizing all labels (consider as one label) and then computing the AUPR, while M-AUPR is computed by first calculating AUPR for each label individually and then performing the unweighted mean of all AUPRs. Both of the metrics can properly represent the overall performance of a multi-label classifier.

In Table 2, the authors show that the overall performance of Graph2GO is good, when compared with a baseline of BLAST. It does seem however, that the BLAST performance is rather high. CAFA typically reports Fmax (not F1) values between 0.4 and 0.5. I am wondering how such high BLAST F1 score was achieved.
One possible issue may be the random split of training and testing sets as reported in P 6 "Comparison with BLAST". A random split does not ensure that there are no homologs for the training set in the test set, which may mean that the authors are testing on part of their training set and test set. The authors should ensure that any sequence in the test set is not too similar to the sequences in the training set.E.g. https://urldefense.proofpoint.com/v2/url?u=https-

3A__www.biorxiv.org_content_10.1101_626507v4.full&d=DwIGaQ&c=k9MF1d71ITtkuJx-PdWme51dKbmfPEvxwt8SFEkBfs4&r=ekJXXuaVVww1UTqb5FGVAqKm1D8j8S7Zredd9hAkY7k&m=0Feecqdx0RHOBGTTz1ex_w6u3ZYkDPiddrOHjdLNzoE&s=NMlQXdy4qrXzxCszFk_3ASxxc-3QmA6j1BxmSisYeLM&e=

Authors' answer: Thanks for pointing out this question. We have added a new comparison situation where all training samples sharing more than 50% identity with one of the test samples are removed. Therefore, we ended up with two datasets: (1) full dataset that uses all randomly split training samples; (2) partial dataset that removes potential homologs. We can see that the results on the partial dataset are now similar to the results from CAFA. From the updated Table 2, we can see that Graph2GO is more robust than BLAST given the fact that Graph2GO shows smaller drop than BLAST from evaluation under full dataset condition to partial dataset condition. As a result, Graph2GO outperforms BLAST even more under the partial dataset condition across all ontologies. The detailed results and analysis are in section "Comparison with BLAST" and Table 2.

Minor comments:

Figure 3 is too small to be legible.

Authors' answer: Thanks for pointing out the problem. We have updated Figure 3 for the updated evaluation metrics and made the figure clearer this time.

Table 3: seemingly low F1 performances, per organism.

Authors' answer: The results of each organism is consistent with the results on human, even for the low F1 score. As we answered to the first point, the low F1 score is due to the definition of our previously used F1 (based only on top three predictions). We have changed this type of F1 score to F-max, as defined in the section "Evaluation metrics". The updated results still seem consistent with the results on human.

Reviewer #2's comments:

This paper presents an approach to predicting protein function that leverages heterogenous sources of knowledge about proteins, including relational information derived from protein interactions and sequence similarity relationships. The authors have clearly presented their computational architecture, which is interesting and as far as I know represents a novel contribution.
However, I have some concerns. My main concerns with this manuscript are (a) a number of (apparent) variations in experimental settings that impeded comparability of results and might suggest some cherry-picking, and (b) the lack of comparison with the tasks and data sets used in the community evaluation CAFA (Critical Assessment of

Function Annotation). Together, it means it is difficult to fully judge the contributions of the work.

Major point 1:

- Few details of the DNN are provided; this should be elaborated.

Authors' answer: Thanks for pointing out this problem. We have added more details about DNN in section "Deep neural network classifier" to make it clearer.

- Is the CNN used in the 'Feature transfer' experiments the same as the DNN of the original experiments? If not, what are the differences, and why is the same algorithm not used for comparability? Is retraining needed in any case, can't the comparison of the sparsity vs non-sparsity group be done as an analysis over the results data? I don't feel that this is explained well.

Authors' answer: Thanks for raising this question. The CNN model used in "Feature transfer" experiment is not the same the DNN we used in the second part of our Graph2GO model. We have added a detailed description of this model in the supplementary material named "Convolutional Neural Networks (CNNs)".

The hypothesis we want to claim here is to show Graph2GO, a model making use of feature transfer within the network, is better at dealing with proteins without sufficient features due to the annotation bias. In other words, we want to show that the performance on the sparsity group is not a lot worse than that of on the non-sparsity group, when using Graph2GO. That's why we use the ratio between the performance on sparsity group and non-sparsity group as a measure. We want to compare with a method without using the concept of feature transfer. Actually, we have many choices, for example, SVM, logistic regression, DNN or CNN. The reason why we chose CNN is that CNN performs better than DNN or SVM in this case since it is good at extracting features. But we can also choose other methods to compare with, no matter the method itself is good or not, since what we care about is the ratio of performance between the sparsity group and non-sparsity group.

The re-training is not needed for Graph2GO in this analysis, since we just analyzed the results data by dividing the test results into two groups and calculated the corresponding metrics.

- In the comparison with deepNF and Mashup, the authors do not explain how the embeddings produced by these methods are used. The authors have explained that these methods produce embeddings that are analogous to the embeddings derived by the authors, but then do not use the same approach in the subsequent stages of the classification process; they use a multi-layer NN for Graph2Go and an SVM for the other two methods. For direct comparability, the overall architecture for learning would be kept the same, and only the input embeddings would be changed. How else can we

know whether the key improvement comes from the embedding approach, or the classification model? The current comparison does not appear to be like-for-like.

Authors' answer: Thanks for pointing out this issue. We previously touched a little bit on these two methods in the fourth paragraph in the "Introduction". We have added a few more descriptions of these two methods.

We previously used SVM for these methods as the classification model because they used SVM in their original implementation. In order to make it more comparable, we have changed their classification model to DNN as well, with the same architecture of Graph2GO. We have updated Figure 3 accordingly with new comparison results, and we have shown that our method outperforms other two methods In CC and MF ontologies, and achieve comparable results in BP ontology.

- In the comparison across species, the authors do not explain their experimental framework sufficiently. Do they control for overlap (very high sequence similarity) with the human training data they have used in developing their model? Do they 'hide'/hold back annotations [all, or just some?] for the proteins in these data sets? Furthermore, they claim "decent" performance (compared to what?) and yet the performance on these data sets is far lower than on the other test data they present. This does not at all 'prove' the robustness; it needs to be explained.

Authors' answer: Thanks for pointing out this problem. In the comparison across species, we are actually performing experiments in each species individually. That's to say, for each species, we download their data, and then performing cross-validation within this species, just like what we did for human dataset. We didn't work in a transfer learning manner, training on human data and test on other species.

By conducting experiments in multiple species, we want to see whether the performance is consistent among all species to show the robustness of our method. We have also included the results on human dataset in the Table 3, and we can see that the performance of these six species are consistently decent, except that the results on *s. cerevisiae* and *bacillus subtilis* in BP ontology are a lot better than those of other species.

Major point 2:

There have been a number of studies over several years that explore methods for automatic assignment of protein function (CAFA4 has just started). The authors cite some of this work (Reference 1) but then go on to claim that homology-based methods are still the norm. That was true 10 years ago, but I am not convinced that it is still true today. Importantly, data sets have been prepared for CAFA, as well as many methods, and the authors clearly need to discuss how their work relates to that work. Indeed, I would suggest that without a direct comparison to the data sets used in CAFA, it is not possible for the authors to claim state-of-the-art performance. BLAST is still a meaningful baseline method to compare with, but it is certainly not representative of the

state-of-the-art.
This comparison may not be entirely feasible given that CAFA test sets are large and only a fraction of them are used for the final evaluation, but IMO it is important to explore this and at least attempt to come close to a similar experimental framework to test the authors' approach. At a minimum, the authors should be using the same metrics for evaluation as CAFA -- perhaps in addition to their own metrics -- to enable meaningful comparison, and of course discussing results on that task in relation to theirs. A comparison that only considers embedding methods is incomplete, in particular given that there are several methods used in CAFA that explicitly support integration of multiple sources of information, including relational information (see [1] for instance).

Authors' answer: Thanks for the great suggestions. For the evaluation metrics, we use similar metrics with CAFA: F-max, macro-AUPR and micro-AUPR. We have added the section "Evaluation metrics" under "Results" to describe the metrics in detail. AUROC is used for term-centric evaluation in CAFA. Here we adopt AUPR instead, since as suggested by Davis and Goadrich (reference 42 in the manuscript), when the dataset is highly skewed, which is the case in this protein function prediction task, AUPR is a more informative metric than AUROC.

Mashup and deepNF are both state-of-the-art network-based embedding methods for predicting protein functions by integrating multiple network information. By comparing with these two methods, we have shown that Graph2GO is among the state-of-the-art network-based embedding methods for protein function prediction. We agree that it is ideal to compare with a larger pool of methods, including integrative methods not based on embedding in CAFA. However, we feel that given the scope of the study, we would like to focus on several methods that are recently published and well received in the community. We would like to continue the development and comparison in the lines of CAFA in the future, which may need our own implementations of CAFA methods that require devotion of more time. We think participating in a large-scale competition like CAFA in the future might be beneficial to our method comparison and development.

In summary, our method not only outperforms Mashup and deepNF in performance, but also exhibits some improvements in the method design. For example, Graph2GO can consider both heterogenous network structural information and protein attributes (including sequences, locations and protein domains) while other two methods only consider network features. Besides, the concept of feature transfer adopted by Graph2GO is advantageous for solving the annotation bias, which is a novel contribution.

On metrics, the use of the groupings of GO terms based on sparsity of annotations is not well justified; I do not understand what this represents, in particular given that the number of proteins annotated to a given GO term is not reflective of any biological reality -- it simply reflects areas of study and our current level of biological understanding. GO is organised hierarchically along semantic/ontological lines, and the authors do not appear to recognise this characteristic. Furthermore, there exists a grouping of GO terms called GO Slims which already seeks to reduce the number of

classes that should be considered [2] (now called subsets:https://urldefense.proofpoint.com/v2/url?u=http-3A__geneontology.org_docs_go-2Dsubset-2Dguide_&d=DwIGaQ&c=k9MF1d71ITtkuJx-PdWme51dKbmfPEvxwt8SFEkBfs4&r=ekJXXuaVVww1UTqb5FGVAqKm1D8j8S7Zredd9hAkY7k&m=0Feecqdx0RHOBGTTz1ex_w6u3ZYkDPiddrOHjdLNzoE&s=J9pWUSCW_9XLhgcFjbPLOw2acB-nA9wZrNl5fqLnx44&e=).

Authors' answer: Thanks for raising this question. We group GO terms based on the sparsity of annotations to assess the impact of annotation bias on function prediction, and to show the performance of our method in more detail. Typically, the more data we have (meaning the annotations are less sparse), the better the function prediction model can perform. By dividing the GO terms in groups with different number of annotated proteins and analyzing the results separately, we can show how our model works when the data is sparse or when the data is abundant.

As for GO Slims, we found that they are grouped mainly based on different species. Since we already analyze the results for each species individually, we feel it is better to stay with the original GO groupings. Thanks for the suggestions.

In addition, why are only the top three predictions considered for evaluation purposes, in calculating F1-score? Having an absolute number, rather than based on confidence/probability etc. requires justification.

Authors' answer: Thanks for pointing out this problem. We have changed this metric to F-max as suggested. The definition is provided in section "Evaluation metrics". Previously we used F1-score based on top three predictions since it was used by Mashup and deepNF, and we adopted F1-score as well for the comparison. Now we use F-max to compare all these methods, which is reasonable and more widely used.

In the methods, the authors do not adequately explain their output representation -- is it framed as a multi-label classification problem, or as a multi-class problem (i.e. can a single input have multiple labels in the output)? Is it just a flat set of GO terms, so that the semantic interdependencies between the terms are not considered? (I later found this detail in the Results; it should come earlier.) It is actually a hierarchical classification problem [3].
This hierarchical nature also interacts with metrics and evaluation; please see [4] and [5]. The authors should consider/discuss their evaluation protocols in this context.

Authors' answer: Thanks for raising this issue. Our task is a multi-label problem, we mentioned that in section "Deep neural network classifier" under "Methods". Our output representation is a flat set of GO terms. In our current implementation, we didn't consider the semantic interdependencies between the terms, which might cause inconsistency between the prediction of leaf GO terms and the corresponding parent GO terms. However, when we generate the dataset for model training and testing, we do assign the parent GO term to the protein when the child GO term is present. We

think this will partially solve the issue of incomplete GO term annotations in the dataset. Besides, since we report a probability in range [0, 1] for each GO term, the users can decide the reliability of the GO term (no matter leaf or parent GO term) by the probability.

We have also added some discussion about how we can treat this as a hierarchical classification problem in the future under the “Discussion” section. In the future, one way to treat it as a hierarchical classification is to add the hierarchical constraints in the output layer to force the predictions of leaf nodes are consistent with the predictions of the corresponding parent nodes. Another potential method is to explicitly add the GO hierarchy into the model, along with our other network components to inform the training process, since we can regard the GO hierarchy as a network. We will leave this integration to the future work.

In the section of "Graph2Go pipeline" the authors mention two alternative architectures. Ideally, they would test both architectures (possibly moving the detail to Supplementary) and indicate which worked better.

Authors’ answer: Thanks for pointing out this problem. We have added the comparison in the supplementary material. From the comparison, we see that the model that trains independent VGAE for each network and combines their embeddings is better than the model that first combines the networks and train one VGAE to obtain the overall embeddings. This result supports our Graph2GO architecture.

On language; the writing is in general very good. There are a few colloquialisms that could be replaced: "plenty of" -> "substantial"; "really good" -> "very good" or "excellent". "noises" should just be "noise". "well suitable" should be "well suited". I found at least one typo "nueral".

Authors’ answer: Thanks for pointing these out. We have corrected the language problems.
