# Supplementary material for: Graph2GO: a multi-modal attributed network embedding method for inferring protein functions
Source: Gigascience. 2020 Aug 8;9(8):giaa081. doi: 10.1093/gigascience/giaa081 (PMC7414417; doi:10.1093/gigascience/giaa081)
Supplement: giaa081_Supplemental_File [file giaa081_supplemental_file.pdf]

Graph2GO: a multi-modal attributed network embedding method for inferring protein functions

Supplementary Materials

Kunjie Fan, Yuanfang Guan, and Yan Zhang

Data Sources

SwissProt: SwissProt is the manually annotated section in UniProtKB, a central hub for the collection of functional information on proteins. We downloaded protein sequence, subcellular location and protein domain (Pfam) information for all human proteins from here, and also downloaded Gene Ontology (GO) annotations for biological process (BP), cellular component (CC) and molecular function (MF). The version we use is release 2018_11. Here is the website link: https://www.uniprot.org/uniprot/?query=reviewed:yes

STRING: STRING is a comprehensive database for Protein-Protein Interaction (PPI), which contains information from multiple sources. STRING scores each interaction by the following seven channels: experiments channel, database channel, textmining channel, coexpression channel, neighborhood channel, fusion channel and co-occurence channel. We downloaded the complete protein network data for human and the version is 10.5. Here is the website link: https://www.string-db.org

Building Networks

Building a PPI Network

Protein-protein interactions (PPIs) are obtained from the STRING database. We first filter out interactions between proteins that are not in our SwissProt dataset, which greatly decreases the size of interactions. We then filter out interactions whose combined score (reported by STRING) are less than 300, which ensures that we only use highly confident interactions. The adjacency matrix is constructed based on the remaining interactions

where zero stands for no interaction and one stands for existing interaction. The dimensions of the adjacency matrix are # of proteins × # of proteins. Since the adjacency matrix is sparse, it is stored in the compressed sparse row (csr) format to accelerate the computation.

Building a Sequence Similarity Network

In order to build a sequence similarity network, we use the BLAST program (specifically BLASTP) to find similar sequences for each protein in our dataset. Specifically, we first use the command makeblastdb to build a local BLAST database using all the sequences in our dataset obtained from SwissProt. Then all the sequences are searched against the local database using blastp program. We specify the E-value parameter to be 1e-4 to filter out less confident alignments. The outfmt parameter is set as 10 so that we can get a comma-separated output file which we use to build the similarity network. Similar to building a PPI network, we construct the adjacency matrix and store it in csr format.

Web Server Description

We deploy a web server for our method using Shiny app so that people can easily use our method to query functions of their interested proteins. Here is the link for our Graph2GO web server: https://integrativeomics.shinyapps.io/graph2go

Our server supports four kinds of input IDs, including “Protein name”, “Gene name”, “UniProt ID” and “STRING ID”. Before search, the threshold needs to be specified, which is used to filter the prediction results. After you click the “Search” button, you will see two parts on the right in less than two seconds. The upper part shows the protein-protein interaction network and sequence similarity network with the query protein in the middle. We only display the top ten neighboring proteins and you can view their attributes (subcellular location and protein domain) by putting the mouse on the node. The lower part lists the prediction results for three ontologies separately, ranked according to the scores provided by the model. You can change the threshold to filter the prediction results dynamically.

We also provide downstream clustering analysis based on the embeddings obtained by our Graph2GO model. Several parameters can be tuned to view the neighborhood of the query protein. Enrichment analysis based on hypergeometric test is also supported. The detailed description about how to use our web server is in the "Tutorial" section on our server website.

Convolutional Neural Networks (CNNs)

CNN is a special kind of Neural Network that is designed for processing images, but has worked very well for many other tasks. There are three main types of layers to build a CNN: convolutional layer, pooling layer and fully-connected layer. The convolutional layer performs convolution operation over the inputs and extract the high-level features. Each convolutional layer initializes several kernels to detect different patterns by convoluting over the entire input space. It is common to insert a pooling layer between successive convolutional layers. The pooling layer can greatly reduce the amounts of parameters to control overfitting by performing maximum or average operations over a fixed size. The max pooling layer is trying to keep the most informative features while throwing away non-essential features to reduce the spatial size of the representation. The most common form of a CNN architecture stacks a few convolutional layers, followed by a pooling layer, and repeats this pattern until the feature representation has been reduced to a small size. Then, it is common to transition to fully-connected layers and the last fully-connected layer holds the output.

In our experiments comparing between the sparsity group and non-sparsity group, we used the most common CNN architecture. Our CNN model uses all three node attributes used in our Graph2GO model as the input features to make it more comparable. The input features are first passed to an embedding layer which provides vector representations of size 128. Then a convolutional layer of 32 filters with size of 128 and a max pooling layer of size 64 are applied. Then the flattened output of the max-pooling layer is passed to a fully-connected layer with sigmoid activation function applied to make predictions. Rmsprop algorithm is used to train the model for 20 iterations with a learning rate of 0.001.

Supplementary Tables

Table S1. Classification of amino acids according to their dipoles and volumes of the side chains based on the CT method.

Class	Amino Acids
C_1	Ala, Gly, Val
C_2	Ile, Leu, Phe, Pro
C_3	Tyr, Met, Thr, Ser
C_4	His, Asn, Gln, Tpr
C_5	Arg, Lys
C_6	Asp, Glu
C_7	Cys

Table S2. Performance between the model where we train independent VGAE for each network and combine their embeddings (refers as "individual") and where we first combine the networks and train one VGAE to obtain the overall embedding (refers as "combined") in terms of M-AUPR, m-AUPR and F-max.

Method	CC			MF			BP		
	M-AUPR	m-AUPR	F-max	M-AUPR	m-AUPR	F-max	M-AUPR	m-AUPR	F-max
individual	0.089	0.217	0.285	0.095	0.150	0.197	0.042	0.086	0.106
combined	0.495	0.458	0.469	0.543	0.528	0.620	0.308	0.247	0.353

Table S3. Performance of CNN and Graph2GO on sparsity group and non-sparsity group of the test set in terms of F-max on different groups of GO terms. Here the "sp" means the sparsity group while "non" means non-sparsity group.

Method	CC			MF			BP		
	11-30	31-100	101-300	11-30	31-100	101-300	11-30	31-100	101-300
CNN (sp)	0.089	0.217	0.285	0.095	0.150	0.197	0.042	0.086	0.106
CNN (non)	0.246	0.244	0.293	0.314	0.303	0.384	0.069	0.101	0.145
Graph2GO (sp)	0.4	0.542	0.491	0.447	0.454	0.526	0.304	0.317	0.378
Graph2GO (non)	0.495	0.458	0.469	0.543	0.528	0.620	0.308	0.247	0.353

Supplementary Figures

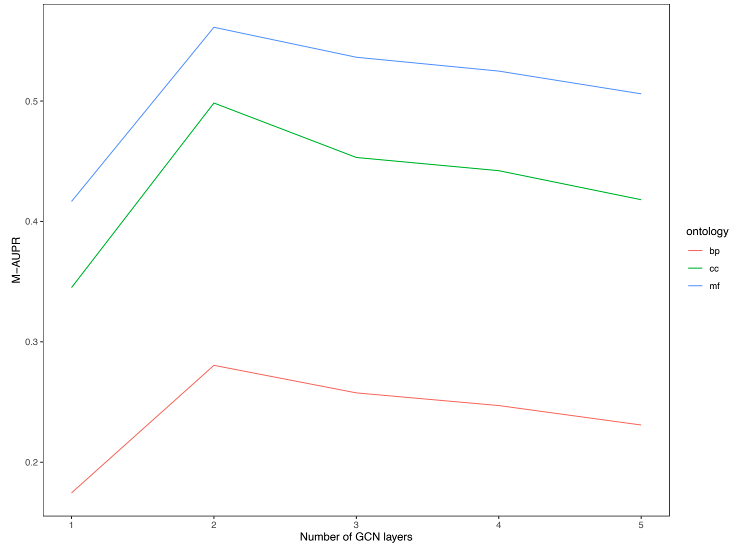

Figure S1: Cross-validation results of choosing different number of layers for GCN model. The performances on all three ontologies reach the best when using two layers of graph convolutional operators. As the number of layers becomes larger, the performance gets worse because applying graph convolutional operator repeatedly may mix of node features from different clusters and make them indistinguishable.

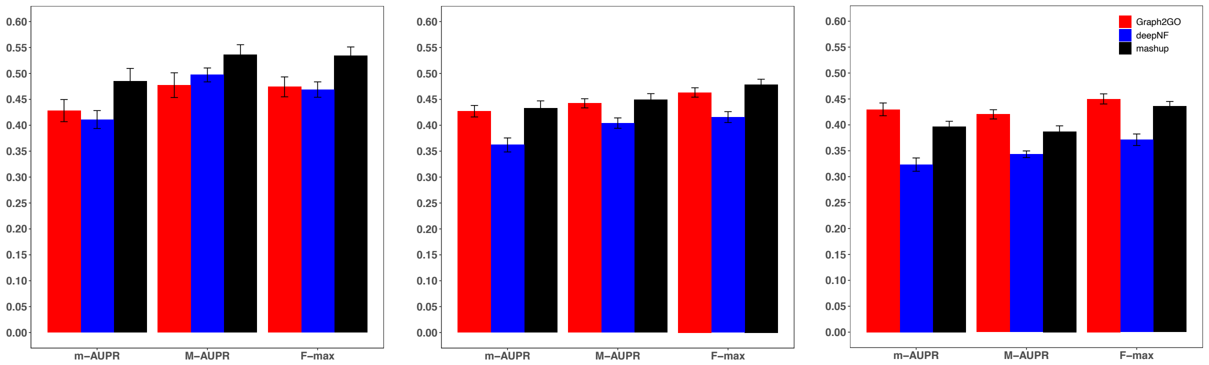

Figure S2: Performance comparison with other state-of-the-art methods in CC ontology for three different sparsity levels. Graph2GO is compared with Mashup and deepNF in terms of three metrics: micro-AUPR, macro-AUPR and F-max. The three barplots show the results of three sparsity levels: [11-30], [31-100] and [101-300]. Each method is

evaluated using 5-fold cross-validation, repeated 10 times to calculate the confidence interval.

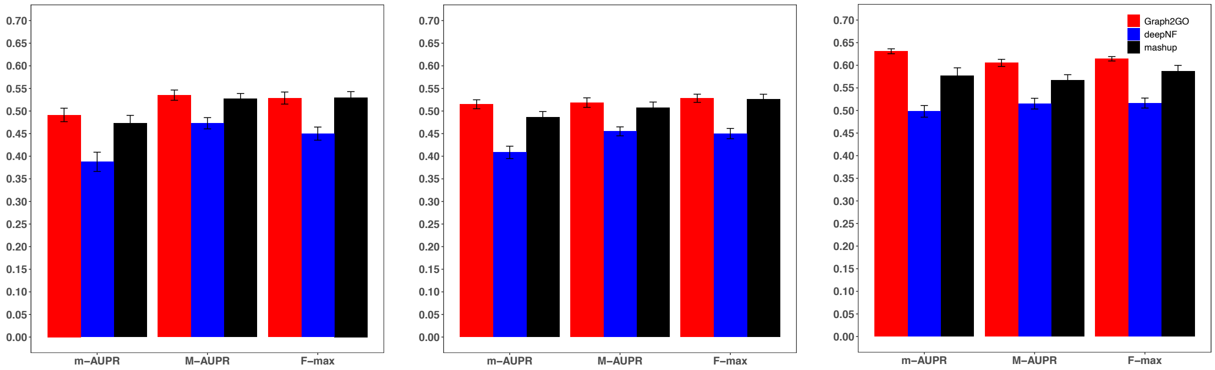

Figure S3: Performance comparison with other state-of-the-art methods in MF ontology for three different sparsity levels. Graph2GO is compared with Mashup and deepNF in terms of three metrics: micro-AUPR, macro-AUPR and F-max. The three barplots show the results of three sparsity levels: [11-30], [31-100] and [101-300]. Each method is evaluated using 5-fold cross-validation, repeated 10 times to calculate the confidence interval.

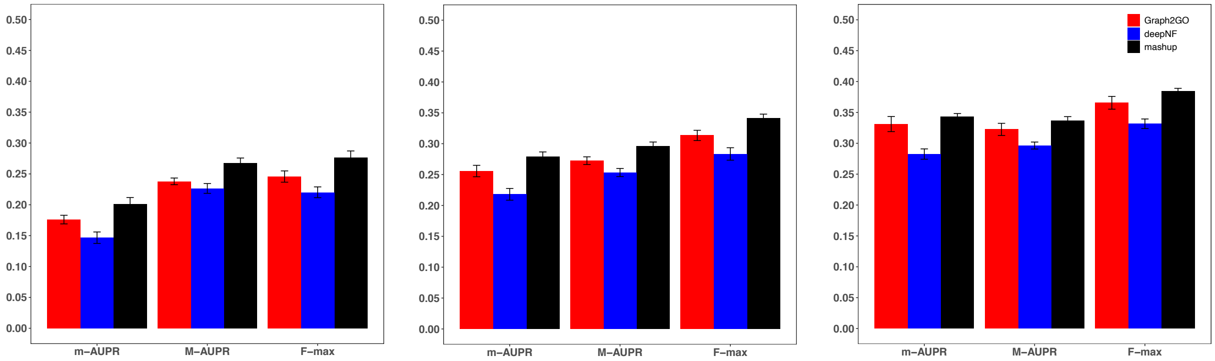

Figure S4: Performance comparison with other state-of-the-art methods in BP ontology for three different sparsity levels. Graph2GO is compared with Mashup and deepNF in terms of three metrics: micro-AUPR, macro-AUPR and F-max. The three barplots show the results of three sparsity levels: [11-30], [31-100] and [101-300]. Each method is

evaluated using 5-fold cross-validation, repeated 10 times to calculate the confidence interval.
