# Supplementary material for: Graph2GO: a multi-modal attributed network embedding method for inferring protein functions
Source: Gigascience. 2020 Aug 8;9(8):giaa081. doi: 10.1093/gigascience/giaa081 (PMC7414417; doi:10.1093/gigascience/giaa081)
Supplement: giaa081_Response_to_Reviewer_Comments_Original_Submission [file giaa081_response_to_reviewer_comments_original_submission.pdf]

Author's Response To Reviewer Comments

Close

Response letter (We also provide a PDF version in the submission)

We thank the editors and reviewers for their insightful comments and suggestions and believe that after addressing each
answers in red color.

> Editor’s comments:
> Both reviewers point out several issues with the evaluation and comparison procedures, which may have introduced bia
further advice from our reviewers and I urge you to make an effort to fully address their comments.

Authors’ answer: We appreciate the feedback. We have worked carefully to solve the problem concerning evaluation and
new evaluation metric F-max that is widely used by the community to replace our previous F1-score that is only based on
include the detailed definition of these evaluation metrics so that general readers can understand. We also elaborate more
as suggested by reviewers. For experiments, we have added more details to clarify any confusions. Besides, we make cha
comparability of the results. We also followed other suggestions carefully and explained how we addressed them in the fol

>Reviewer #1’s comments:
>
>This work describes a new method that uses attributed network representation that uses both interaction networks and
represented as GO terms. This method of integrating heterogeneous information is, to my knowledge, novel and interestin
comments below).

>Major comments:
>My main concern is the rather low F1 scores when compared with the apparent better performance of the AUPR scores,
show average precision and recall so that the reader can establish the contribution of the precision and recall to the F1 sc
BP). I believe the authors could explain that, especially in light of the overall good performance in Table 2. Again, Figure 3
work with the F1 of 0.717 reported in Table 2?

Authors’ answer: Thanks for pointing out this problem. We should apologize for the inconsistence of the use of F1-score. I
based on top three predictions, as used by Mashup and deepNF that we want to compare with. In Table 2, when comparin
based on top three predictions cannot be calculated for BLAST. The F1-score based on top three predictions tend to be sm
the inconsistence, we decide to change the evaluation metric to F-max, the maximum F1-score over all possible threshold
Assessment of protein Function Annotation algorithms). The detailed definition of this metric along with others are introdu
results based on F-max, the performance is more consistent with other metrics, and our model is still better than other m

>I could not find definitions for m-AUPR and M-AUPR. It is my understanding that micro-averaged area under the precisio
computing the AUPR; Macro-AUPR (M-AUPR) is computed by first computing the AUPR for each function separately, and th
know how this was implemented here, and how the differences in performance are explained. While these performance m
I think that the authors should elaborate a bit more on the choice of these metrics and their meaning, as Gigascience aim
learning.

Authors’ answer: Thanks for pointing out this issue. We have added a new section “Evaluation metric” under “Results” to
prediction is a multi-label task, we need to evaluate the performance on all labels (GO terms). One of the most popular m
operating characteristic (ROC) curves. However, given the fact that the dataset is kind of skewed (more negative samples
informative picture of an algorithm’s performance than ROC curves, as pointed by Davis and Goadrich (reference 42 in the
macro-AUPR (M-AUPR) as two evaluation metrics. m-AUPR is computed by first vectorizing all labels (consider as one labe
first calculating AUPR for each label individually and then performing the unweighted mean of all AUPRs. Both of the metri
classifier.

>In Table 2, the authors show that the overall performance of Graph2GO is good, when compared with a baseline of BLAS
high. CAFA typically reports Fmax (not F1) values between 0.4 and 0.5. I am wondering how such high BLAST F1 score wa
One possible issue may be the random split of training and testing sets as reported in P 6 "Comparison with BLAST". A ran
training set in the test set, which may mean that the authors are testing on part of their training set and test set. The aut

similar to the sequences in the training set.E.g. https://urldefense.proofpoint.com/v2/url?u=https-3A__www.biorxiv.org_c
PdWme51dKbmfPEvxwt8SFEkBfs4&r=ekJXXuaVVww1UTqb5FGVAqKm1D8j8S7Zredd9hAkY7k&m=0Feecqdx0RHOBGTTz1e
3QmA6j1BxmSisYeLM&e=

Authors' answer: Thanks for pointing out this question. We have added a new comparison situation where all training sam
are removed. Therefore, we ended up with two datasets: (1) full dataset that uses all randomly split training samples; (2)
the results on the partial dataset are now similar to the results from CAFA. From the updated Table 2, we can see that Gra
shows smaller drop than BLAST from evaluation under full dataset condition to partial dataset condition. As a result, Grap
condition across all ontologies. The detailed results and analysis are in section "Comparison with BLAST" and Table 2.

>Minor comments:
>
>Figure 3 is too small to be legible.

Authors' answer: Thanks for pointing out the problem. We have updated Figure 3 for the updated evaluation metrics and r

>Table 3: seemingly low F1 performances, per organism.

Authors' answer: The results of each organism is consistent with the results on human, even for the low F1 score. As we a
of our previously used F1 (based only on top three predictions). We have changed this type of F1 score to F-max, as defin
seem consistent with the results on human.

>Reviewer #2's comments:
>
>This paper presents an approach to predicting protein function that leverages heterogenous sources of knowledge about
interactions and sequence similarity relationships. The authors have clearly presented their computational architecture, wh
contribution.
However, I have some concerns. My main concerns with this manuscript are (a) a number of (apparent) variations in expe
suggest some cherry-picking, and (b) the lack of comparison with the tasks and data sets used in the community evaluati
means it is difficult to fully judge the contributions of the work.
>
>Major point 1:
>
>- Few details of the DNN are provided; this should be elaborated.

Authors' answer: Thanks for pointing out this problem. We have added more details about DNN in section "Deep neural ne

>- Is the CNN used in the 'Feature transfer' experiments the same as the DNN of the original experiments? If not, what ar
comparability? Is retraining needed in any case, can't the comparison of the sparsity vs non-sparsity group be done as an
well.

Authors' answer: Thanks for raising this question. The CNN model used in "Feature transfer" experiment is not the same t
have added a detailed description of this model in the supplementary material named "Convolutional Neural Networks (CN

The hypothesis we want to claim here is to show Graph2GO, a model making use of feature transfer within the network, is
the annotation bias. In other words, we want to show that the performance on the sparsity group is not a lot worse than t
why we use the ratio between the performance on sparsity group and non-sparsity group as a measure. We want to comp
Actually, we have many choices, for example, SVM, logistic regression, DNN or CNN. The reason why we chose CNN is tha
good at extracting features. But we can also choose other methods to compare with, no matter the method itself is good o
between the sparsity group and non-sparsity group.

The re-training is not needed for Graph2GO in this analysis, since we just analyzed the results data by dividing the test re

>- In the comparison with deepNF and Mashup, the authors do not explain how the embeddings produced by these metho
produce embeddings that are analogous to the embeddings derived by the authors, but then do not use the same approac
multi-layer NN for Graph2Go and an SVM for the other two methods. For direct comparability, the overall architecture for
would be changed. How else can we know whether the key improvement comes from the embedding approach, or the clas
like-for-like.

Authors' answer: Thanks for pointing out this issue. We previously touched a little bit on these two methods in the fourth
descriptions of these two methods.

We previously used SVM for these methods as the classification model because they used SVM in their original implement
classification model to DNN as well, with the same architecture of Graph2GO. We have updated Figure 3 accordingly with
outperforms other two methods In CC and MF ontologies, and achieve comparable results in BP ontology.

>- In the comparison across species, the authors do not explain their experimental framework sufficiently. Do they contro
training data they have used in developing their model? Do they 'hide'/hold back annotations [all, or just some?] for the p
performance (compared to what?) and yet the performance on these data sets is far lower than on the other test data the
be explained.

Authors' answer: Thanks for pointing out this problem. In the comparison across species, we are actually performing expe
species, we download their data, and then performing cross-validation within this species, just like what we did for humar
human data and test on other species.

By conducting experiments in multiple species, we want to see whether the performance is consistent among all species t
results on human dataset in the Table 3, and we can see that the performance of these six species are consistently decen
ontology are a lot better than those of other species.

>Major point 2:
>
>There have been a number of studies over several years that explore methods for automatic assignment of protein funct
(Reference 1) but then go on to claim that homology-based methods are still the norm. That was true 10 years ago, but I
have been prepared for CAFA, as well as many methods, and the authors clearly need to discuss how their work relates to
comparison to the data sets used in CAFA, it is not possible for the authors to claim state-of-the-art performance. BLAST i
certainly not representative of the state-of-the-art.
This comparison may not be entirely feasible given that CAFA test sets are large and only a fraction of them are used for t
least attempt to come close to a similar experimental framework to test the authors' approach. At a minimum, the author
perhaps in addition to their own metrics -- to enable meaningful comparison, and of course discussing results on that task
methods is incomplete, in particular given that there are several methods used in CAFA that explicitly support integration
(see [1] for instance).

Authors' answer: Thanks for the great suggestions. For the evaluation metrics, we use similar metrics with CAFA: F-max,
"Evaluation metrics" under "Results" to describe the metrics in detail. AUROC is used for term-centric evaluation in CAFA.
Goadrich (reference 42 in the manuscript), when the dataset is highly skewed, which is the case in this protein function p

Mashup and deepNF are both state-of-the-art network-based embedding methods for predicting protein functions by integ
methods, we have shown that Graph2GO is among the state-of-the-art network-based embedding methods for protein fu
pool of methods, including integrative methods not based on embedding in CAFA. However, we feel that given the scope c
recently published and well received in the community. We would like to continue the development and comparison in the
implementations of CAFA methods that require devotion of more time. We think participating in a large-scale competition
comparison and development.

In summary, our method not only outperforms Mashup and deepNF in performance, but also exhibits some improvements
heterogenous network structural information and protein attributes (including sequences, locations and protein domains)
the concept of feature transfer adopted by Graph2GO is advantageous for solving the annotation bias, which is a novel co

>On metrics, the use of the groupings of GO terms based on sparsity of annotations is not well justified; I do not underst
proteins annotated to a given GO term is not reflective of any biological reality -- it simply reflects areas of study and our
hierarchically along semantic/ontological lines, and the authors do not appear to recognise this characteristic. Furthermor
already seeks to reduce the number of classes that should be considered [2] (now called subsets:https://urldefense.proof
2Dsubset-2Dguide_&d=DwIGaQ&c=k9MF1d71ITtkuJx-
PdWme51dKbmfPEvxwt8SFEkBfs4&r=ekJXXuaVVww1UTqb5FGVAqKm1D8j8S7Zredd9hAkY7k&m=0Feecqdx0RHOBGTTz1e
nA9wZrNl5fqLnx44&e=).

Authors' answer: Thanks for raising this question. We group GO terms based on the sparsity of annotations to assess the
performance of our method in more detail. Typically, the more data we have (meaning the annotations are less sparse), t
the GO terms in groups with different number of annotated proteins and analyzing the results separately, we can show ho
abundant.

As for GO Slims, we found that they are grouped mainly based on different species. Since we already analyze the results f
original GO groupings. Thanks for the suggestions.

>In addition, why are only the top three predictions considered for evaluation purposes, in calculating F1-score? Having a
etc. requires justification.

Authors' answer: Thanks for pointing out this problem. We have changed this metric to F-max as suggested. The definitio
F1-score based on top three predictions since it was used by Mashup and deepNF, and we adopted F1-score as well for th
which is reasonable and more widely used.

>In the methods, the authors do not adequately explain their output representation -- is it framed as a multi-label classifi
have multiple labels in the output)? Is it just a flat set of GO terms, so that the semantic interdependencies between the t
should come earlier.) It is actually a hierarchical classification problem [3].
This hierarchical nature also interacts with metrics and evaluation; please see [4] and [5]. The authors should consider/di

Authors' answer: Thanks for raising this issue. Our task is a multi-label problem, we mentioned that in section "Deep neur
is a flat set of GO terms. In our current implementation, we didn't consider the semantic interdependencies between the t
leaf GO terms and the corresponding parent GO terms. However, when we generate the dataset for model training and te
child GO term is present. We think this will partially solve the issue of incomplete GO term annotations in the dataset. Bes
term, the users can decide the reliability of the GO term (no matter leaf or parent GO term) by the probability.

We have also added some discussion about how we can treat this as a hierarchical classification problem in the future und
hierarchical classification is to add the hierarchical constraints in the output layer to force the predictions of leaf nodes are
Another potential method is to explicitly add the GO hierarchy into the model, along with our other network components t
hierarchy as a network. We will leave this integration to the future work.

>In the section of "Graph2Go pipeline" the authors mention two alternative architectures. Ideally, they would test both ar
indicate which worked better.

Authors' answer: Thanks for pointing out this problem. We have added the comparison in the supplementary material. Fro
VGAE for each network and combines their embeddings is better than the model that first combines the networks and trai
our Graph2GO architecture.

>On language; the writing is in general very good. There are a few colloquialisms that could be replaced: "plenty of" -> "s
should just be "noise". "well suitable" should be "well suited". I found at least one typo "nueral".

Authors' answer: Thanks for pointing these out. We have corrected the language problems.

Close
