# Supplementary material for: Graph2GO: a multi-modal attributed network embedding method for inferring protein functions
Source: Gigascience. 2020 Aug 8;9(8):giaa081. doi: 10.1093/gigascience/giaa081 (PMC7414417; doi:10.1093/gigascience/giaa081)
Supplement: giaa081_Response_to_Reviewer_Comments_Revision_1 [file giaa081_response_to_reviewer_comments_revision_1.pdf]

Author's Response To Reviewer Comments

Close

We have made the edits to the final manuscript. Please refer to Gigascience_Graph2GO_v2.pdf
We have also submited the latex package containing all the things to generate the pdf without highlight color.

Close
