# Supplementary material for: Graph2GO: a multi-modal attributed network embedding method for inferring protein functions
Source: Gigascience. 2020 Aug 8;9(8):giaa081. doi: 10.1093/gigascience/giaa081 (PMC7414417; doi:10.1093/gigascience/giaa081)
Supplement: giaa081_Reviewer_1_Report_Original_Submission — Iddo Friedberg -- 1/21/2020 Reviewed [file giaa081_reviewer_1_report_original_submission.pdf]

Reviewer Report

Title: Graph2GO: a multi-modal attributed network embedding method for inferring protein functions

Version: Original Submission **Date:** 1/21/2020

Reviewer name: Iddo Friedberg

Reviewer Comments to Author:

This work describes a new method that uses attributed network representation that uses both interaction networks and protein features to predict protein function, when the function are represented as GO terms. This method of integrating heterogeneous information is, to my knowledge, novel and interesting. The methodology and results are mostly well described (but see comments below).

Major comments:

My main concern is the rather low F1 scores when compared with the apparent better performance of the AUPR scores, as shown in Table 1. (Incidentally, of showing F1 scores, why not show average precision and recall so that the reader can establish the contribution of the precision and recall to the F1 score?) The F1 scores are quite low (0.29 for CC and MF, and 0.07 for BP). I believe the authors could explain that, especially in light of the overall good performance in Table 2. Again, Figure 3 shows a low performance of F1 (0.25-0.35) of F1. How does that work with the F1 of 0.717 reported in Table 2?

I could not find definitions for m-AUPR and M-AUPR. It is my understanding that micro-averaged area under the precision-recall curve (m-AUPR) is computed by first vectorizing and then computing the AUPR; Macro-AUPR (M-AUPR) is computed by first computing the AUPR for each function separately, and then averaging these values across all functions. I would like to know how this was implemented here, and how the differences in performance are explained. While these performance metrics may be well-understood in the machine learning community, I think that the authors should elaborate a bit more on the choice of these metrics and their meaning, as Gigascience aims at a broader audience than those well-versed in machine learning.

In Table 2, the authors show that the overall performance of Graph2GO is good, when compared with a baseline of BLAST. It does seem however, that the BLAST performance is rather high. CAFA typically reports Fmax (not F1) values between 0.4 and 0.5. I am wondering how such high BLAST F1 score was achieved.

One possible issue may be the random split of training and testing sets as reported in P 6 "Comparison with BLAST". A random split does not ensure that there are no homologs for the training set in the tet set, which may mean that the authors are testing on part of their training set and test set. The authors should ensure that any sequence in the test set is not too similar to the sequences in the training set.E.g. https://www.biorxiv.org/content/10.1101/626507v4.full

Minor comments:

Figure 3 is too small to be legible.

Table 3: seemingly low F1 performances, per organism.

Level of Interest

Please indicate how interesting you found the manuscript: Choose an item.

Quality of Written English

Please indicate the quality of language in the manuscript: Choose an item.

Declaration of Competing Interests

Please complete a declaration of competing interests, considering the following questions:

- Have you in the past five years received reimbursements, fees, funding, or salary from an organisation that may in any way gain or lose financially from the publication of this manuscript, either now or in the future?
- Do you hold any stocks or shares in an organisation that may in any way gain or lose financially from the publication of this manuscript, either now or in the future?
- Do you hold or are you currently applying for any patents relating to the content of the manuscript?
- Have you received reimbursements, fees, funding, or salary from an organization that holds or has applied for patents relating to the content of the manuscript?
- Do you have any other financial competing interests?
- Do you have any non-financial competing interests in relation to this paper?

If you can answer no to all of the above, write 'I declare that I have no competing interests' below. If your reply is yes to any, please give details below.

I declare that I have no competing interests

I agree to the open peer review policy of the journal. I understand that my name will be included on my report to the authors and, if the manuscript is accepted for publication, my named report including any attachments I upload will be posted on the website along with the authors' responses. I agree for my report to be made available under an Open Access Creative Commons CC-BY license (http://creativecommons.org/licenses/by/4.0/). I understand that any comments which I do not wish to be included in my named report can be included as confidential comments to the editors, which will not be published.

Choose an item.

To further support our reviewers, we have joined with Publons, where you can gain additional credit to further highlight your hard work (see: https://publons.com/journal/530/gigascience). On publication of this paper, your review will be automatically added to Publons, you can then choose whether or not to claim your Publons credit. I understand this statement.

Yes Choose an item.
