# Supplementary material for: Graph2GO: a multi-modal attributed network embedding method for inferring protein functions
Source: Gigascience. 2020 Aug 8;9(8):giaa081. doi: 10.1093/gigascience/giaa081 (PMC7414417; doi:10.1093/gigascience/giaa081)
Supplement: giaa081_Reviewer_2_Report_Original_Submission — Karin Verspoor -- 1/24/2020 Reviewed [file giaa081_reviewer_2_report_original_submission.pdf]

Reviewer Report

Title: Graph2GO: a multi-modal attributed network embedding method for inferring protein functions

Version: Original Submission **Date:** 1/24/2020

Reviewer name: Karin Verspoor

Reviewer Comments to Author:

This paper presents an approach to predicting protein function that leverages heterogenous sources of knowledge about proteins, including relational information derived from protein interactions and sequence similarity relationships. The authors have clearly presented their computational architecture, which is interesting and as far as I know represents a novel contribution.
However, I have some concerns. My main concerns with this manuscript are (a) a number of (apparent) variations in experimental settings that impeded comparability of results and might suggest some cherry-picking, and (b) the lack of comparison with the tasks and data sets used in the community evaluation CAFA (Critical Assessment of Function Annotation). Together, it means it is difficult to fully judge the contributions of the work.
On the first point:
- Few details of the DNN are provided; this should be elaborated.
- Is the CNN used in the 'Feature transfer' experiments the same as the DNN of the original experiments? If not, what are the differences, and why is the same algorithm not used for comparability? Is retraining needed in any case, can't the comparison of the sparsity vs non-sparsity group be done as an analysis over the results data? I don't feel that this is explained well.
- In the comparison with deepNF and Mashup, the authors do not explain how the embeddings produced by these methods are used. The authors have explained that these methods produce embeddings that are analogous to the embeddings derived by the authors, but then do not use the same approach in the subsequent stages of the classification process; they use a multi-layer NN for Graph2Go and an SVM for the other two methods. For direct comparability, the overall architecture for learning would be kept the same, and only the input embeddings would be changed. How else can we know whether the key improvement comes from the embedding approach, or the classification model? The current comparison does not appear to be like-for-like.
- In the comparison across species, the authors do not explain their experimental framework sufficiently. Do they control for overlap (very high sequence similarity) with the human training data they have used in developing their model? Do they 'hide'/hold back annotations [all, or just some?] for the proteins in these data sets? Furthermore, they claim "decent" performance (compared to what?) and yet the performance on these data sets is far lower than on the other test data they present. This does not at all 'prove' the robustness; it needs to be explained.
On the second point:
There have been a number of studies over several years that explore methods for automatic assignment of protein function (CAFA4 has just started). The authors cite some of this work (Reference 1) but then

go on to claim that homology-based methods are still the norm. That was true 10 years ago, but I am not convinced that it is still true today. Importantly, data sets have been prepared for CAFA, as well as many methods, and the authors clearly need to discuss how their work relates to that work. Indeed, I would suggest that without a direct comparison to the data sets used in CAFA, it is not possible for the authors to claim state-of-the-art performance. BLAST is still a meaningful baseline method to compare with, but it is certainly not representative of the state-of-the-art.

This comparison may not be entirely feasible given that CAFA test sets are large and only a fraction of them are used for the final evaluation, but IMO it is important to explore this and at least attempt to come close to a similar experimental framework to test the authors' approach. At a minimum, the authors should be using the same metrics for evaluation as CAFA -- perhaps in addition to their own metrics -- to enable meaningful comparison, and of course discussing results on that task in relation to theirs. A comparison that only considers embedding methods is incomplete, in particular given that there are several methods used in CAFA that explicitly support integration of multiple sources of information, including relational information (see [1] for instance).

On metrics, the use of the groupings of GO terms based on sparsity of annotations is not well justified; I do not understand what this represents, in particular given that the number of proteins annotated to a given GO term is not reflective of any biological reality -- it simply reflects areas of study and our current level of biological understanding. GO is organised hierarchically along semantic/ontological lines, and the authors do not appear to recognise this characteristic. Furthermore, there exists a grouping of GO terms called GO Slims which already seeks to reduce the number of classes that should be considered [2] (now called subsets: http://geneontology.org/docs/go-subset-guide/).

In addition, why are only the top three predictions considered for evaluation purposes, in calculating F1-score? Having an absolute number, rather than based on confidence/probability etc. requires justification.

In the methods, the authors do not adequately explain their output representation -- is it framed as a multi-label classification problem, or as a multi-class problem (i.e. can a single input have multiple labels in the output)? Is it just a flat set of GO terms, so that the semantic interdependencies between the terms are not considered? (I later found this detail in the Results; it should come earlier.) It is actually a hierarchical classification problem [3].

This hierarchical nature also interacts with metrics and evaluation; please see [4] and [5]. The authors should consider/discuss their evaluation protocols in this context.

In the section of "Graph2Go pipeline" the authors mention two alternative architectures. Ideally, they would test both architectures (possibly moving the detail to Supplementary) and indicate which worked better.

On language; the writing is in general very good. There are a few colloquialisms that could be replaced: "plenty of" -> "substantial"; "really good" -> "very good" or "excellent". "noises" should just be "noise". "well suitable" should be "well suited". I found at least one typo "nueral".

[1] Sokolov et al (2013) Combining Heterogeneous Data Sources for Accurate Functional Annotation of Proteins. BMC Bioinformatics 14(S3):S10, special issue on CAFA. [PMID:23514123]

[2] Rhee, Wood, Dolinksi, Draghici (2008) Use and misuse of the gene ontology annotations; Nature Reviews Genetic s9, pages509-515 https://doi.org/10.1038/nrg2363

[3] Kahanda I, Funk C, Khattak F, Verspoor KM, Ben-Hur A. (2015) A close look at protein function

prediction evaluation protocols. Gigascience 4:41. doi:10.1186/s13742-015-0082-5 [PMID:26380075]
[4] Clark, Radivojac (2013) Vol. 29 ISMB/ECCB 2013, pages i53-i61 Bioinformatics doi:10.1093/bioinformatics/btt228
[5] Verspoor et al (2006). A Categorization Approach to Automated Ontological Function Annotation. Protein Science, v.15, pp.1544-1549. http://www.ncbi.nlm.nih.gov/pmc/articles/PMC2242540/ ("Evaluation measures" section)

Level of Interest

Please indicate how interesting you found the manuscript: Choose an item.

Quality of Written English

Please indicate the quality of language in the manuscript: Choose an item.

Declaration of Competing Interests

Please complete a declaration of competing interests, considering the following questions:

- Have you in the past five years received reimbursements, fees, funding, or salary from an organisation that may in any way gain or lose financially from the publication of this manuscript, either now or in the future?
- Do you hold any stocks or shares in an organisation that may in any way gain or lose financially from the publication of this manuscript, either now or in the future?
- Do you hold or are you currently applying for any patents relating to the content of the manuscript?
- Have you received reimbursements, fees, funding, or salary from an organization that holds or has applied for patents relating to the content of the manuscript?
- Do you have any other financial competing interests?
- Do you have any non-financial competing interests in relation to this paper?

If you can answer no to all of the above, write 'I declare that I have no competing interests' below. If your reply is yes to any, please give details below.

I declare that I have no competing interests.

I agree to the open peer review policy of the journal. I understand that my name will be included on my report to the authors and, if the manuscript is accepted for publication, my named report including any attachments I upload will be posted on the website along with the authors' responses. I agree for my report to be made available under an Open Access Creative Commons CC-BY license (http://creativecommons.org/licenses/by/4.0/). I understand that any comments which I do not wish to

be included in my named report can be included as confidential comments to the editors, which will not be published.

Choose an item.

To further support our reviewers, we have joined with Publons, where you can gain additional credit to further highlight your hard work (see: https://publons.com/journal/530/gigascience). On publication of this paper, your review will be automatically added to Publons, you can then choose whether or not to claim your Publons credit. I understand this statement.

Yes Choose an item.
